# Comparison of Micelles Single- and Dual-Targeted with Folic Acid and Biotin as the Delivery System of Docetaxel—The Influence of the Type and Amount of the Ligand on Morphology, Physicochemical Properties, and Cytotoxicity

**DOI:** 10.1021/acs.molpharmaceut.5c00215

**Published:** 2025-06-26

**Authors:** Magdalena Jurczyk, Ryszard Smolarczyk, Monika Musiał-Kulik, Joanna Ciepła, Sybilla Matuszczak, Tomasz Cichoń, Justyna Czapla, Marcelina Bochenek, Aleksander Foryś, Dorota Wrześniok, Artur Beberok, Katarzyna Jelonek

**Affiliations:** † Department of Pharmaceutical Chemistry, Faculty of Pharmaceutical Sciences in Sosnowiec, 49613 Medical University of Silesia, Jagiellońska 4, 41-200 Sosnowiec, Poland; ‡ Centre of Polymer and Carbon Materials, 49559 Polish Academy of Sciences, Curie-Skłodowska 34 Street, 41-819 Zabrze, Poland; § Center for Translational Research and Molecular Biology of Cancer, 49598 Maria Skłodowska-Curie National Research Institute of Oncology, Gliwice Branch, Wybrzeże Armii Krajowej Street 15, 44-102 Gliwice, Poland

**Keywords:** dual-targeted micelles, folic acid, biotin, docetaxel, PLA−PEG, active targeting, nanoparticles

## Abstract

Nanoparticles (NPs) dual-targeted with folic acid (FA) and biotin (BIO) are developed to overcome problems associated with conventional chemotherapy and tumor heterogeneity. Although some preliminary studies have been conducted to develop dual-targeted micelles for delivery of docetaxel (Dtx), a more detailed analysis is needed to discover their pharmaceutical potential. Therefore, this manuscript is focused on a comprehensive analysis of the influence of the type and density of targeting ligand on micelles’ morphology, physicochemical properties, drug loading and release, cell internalization, cytotoxicity in vitro against several kinds of cells and in vivo on two mice cancer models. For this purpose, three kinds of micelles were obtained: decorated with one type of ligand (FA or BIO) and dual-targeted. The micelles contained different amounts of FA- and/or BIO-functionalized polymer (10, 25, and 50%). Additionally, the dual-targeted micelles were compared with a mixture of two kinds of single-targeted micelles. The study showed that the cytotoxic effect of micelles strongly depends on the type of cancer cells and their surface characteristics. The micelles with a higher density of ligands may have higher efficiency against cells with lower expression of cell surface receptors (SK-BR-3 and MCF-7). Conversely, micelles with lower ligand density may be advantageous against cancer cells with overexpression of surface receptors (HeLa, 4T1). Interestingly, the drug delivered in FA-targeted micelles showed higher cytotoxicity compared to dual-targeted micelles in which two ligands are present on the same NP. However, Dtx delivered in a mixture of micelles single-targeted with FA and BIO showed the highest cytotoxic effect, which was confirmed in vitro and in vivo on the mice model.

## Graphical abstract

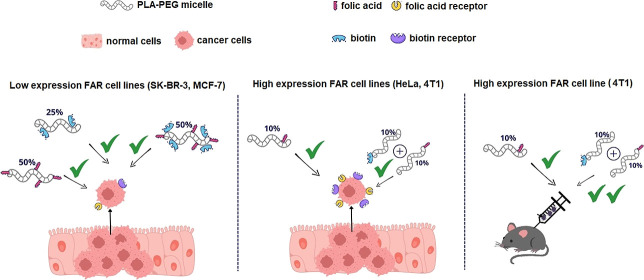

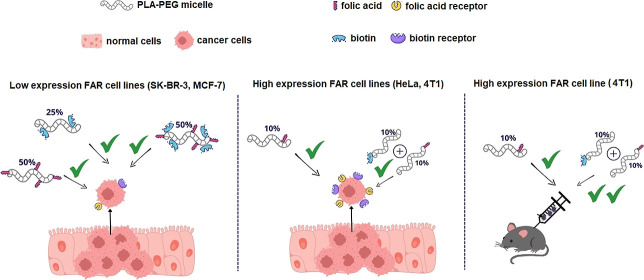

## 1 Introduction

Cancer represents one of the major causes of death worldwide, and thus, there is a need for the development of new therapeutic strategies. Although the therapy of solid tumors is based on surgery, radiotherapy, and chemotherapy,^1^ chemotherapy remains the only available treatment option in the cases that eliminate the possibility of surgical treatment, such as metastasized tumors or lesions. However, conventional chemotherapy may result in many severe side effects due to unspecific targeting to the tumor, which causes accumulation of the drug in healthy tissue and difficulties in achieving therapeutic levels of the drug in the tumor, severe side effects, and toxicity limiting the drug dose.^1,2^ The example of the drug for which toxicity and its systemic side effects limit the effectiveness of treatment is docetaxel (Dtx). It belongs to the second generation of the taxoid family that shows anticancer potency against numerous cancers, e.g., ovarian, breast, nonsmall cell lung, gastric, and prostate.^3,4^ Dtx inhibits the depolymerization of microtubules that causes cell cycle arrest in the G2/M phase and cell death. It also upregulates cell cycle inhibitor p27 expression and downregulates antiapoptotic gene Bcl-2 expression.^3,5^

To overcome the limitations associated with conventional chemotherapy, nanoparticles (NPs) and nanotechnology are gaining interest in cancer therapy because they have the potential to provide more effective and targeted drug delivery.^6^ Biodegradable polymeric micelles represent nano-sized colloidal systems that have gained recognition as highly promising platforms for targeted anticancer drug delivery, attributed to their superior biocompatibility, extended circulation half-life, preferential tumor accumulation, and in vivo degradability.^7–9^ They possess the ability to solubilize hydrophobic pharmaceutical compounds in the core, while the outer hydrophilic corona provides both biocompatibility and extended plasma residence time by avoiding rapid reticuloendothelial clearance.^10,11^ Among amphiphilic copolymers used for cancer drug delivery, poly­(lactic acid)–poly­(ethylene glycol) (PLA–PEG) has attracted considerable interest.^12^ Due to its biocompatibility, extended circulation half-life, and capacity for simple functionalization with various ligands, poly­(ethylene glycol) (PEG) is regarded as one of the most promising carriers for tumor targeting.^12^ Among hydrophobic polymer blocks, poly­(lactide) (PLA) is widely utilized because of its biocompatibility, biodegradation through hydrolytic and enzymatic mechanisms, and extensive range of mechanical and physical properties that can be tailored for various applications.^11–13^

Passive tumor targeting via the enhanced permeability and retention (EPR) effect represents the most common mechanism for nanomedicine-based anticancer therapy. However, many studies have indicated that the EPR effect may not provide an effective distribution of micelles. Therefore, the most recent findings suggest that future nanomedicine development may need novel design principles emphasizing active nanocarrier targeting.^1,14–16^ Ligand–receptor recognition may facilitate efficient drug delivery into tumor cells via receptor-mediated endocytosis, consequently exhibiting an enhanced therapeutic efficacy relative to passive targeting strategies in cancer therapy. A variety of small molecules, including folic acid (FA) and biotin (BIO), have been utilized as targeting ligands in cancer chemotherapy because of their advantageous characteristics: high stability, low production costs, and ease of handling.^17,18^ FA (vitamin B_9_) is a synthetic form of naturally occurring folate and presents high affinity and specificity for folic acid receptors (FAR). Overexpression of FAR that is greater by 100–300 times in comparison to normal tissues has been confirmed for various carcinomas, e.g., ovarian, renal, pancreatic, lung, breast, and brain.^18–22^ Biotin (vitamin B_7_) is a water-soluble molecule that is critical for cell division, growth, fatty acid biosynthesis, fat and amino acid metabolism, and cellular energy production. Therefore, BIO can be selectively recognized by sodium-dependent multivitamin transporters (SMVT) that are typically overexpressed in rapidly proliferating malignant cells.^17,18,23^

Nevertheless, despite advances, single-ligand nanomedicines may demonstrate limited efficacy due to tumor microenvironment complexity. Tumor heterogeneity exists across multiple levels—between patients, between primary and metastatic lesions, and within individual tumors—thereby presenting a substantial challenge for the development of effective therapy strategies.^24^ Within heterogeneous tumors, diverse cellular populations exist, with each clonal subpopulation exhibiting differences in molecular target expression, quantity, and quality characteristics (accessibility and affinity). Vitamin receptor levels may vary significantly between cells within the tumor. Consequently, the response to targeted therapy within a tumor can substantially vary.^25,26^ Active targeting relies on ligand–receptor interactions at the tumor cell surface, making therapeutic efficacy dependent on receptor expression levels that may fluctuate throughout cancer progression.^27^ Moreover, the binding of ligands to receptors follows saturable kinetics and involves dual processes: receptor recycling and new receptor biosynthesis.^14^ It was also reported that various receptors are often upregulated on tumor cells, and drug resistance may be caused by upregulation of alternative receptors and switching between two receptors.^28^ These factors may affect the delivery of single-ligand nanocarriers and reduce the drug efficacy. The presence of two targeting ligands on nanomedicine can enhance their tumor-specific selectivity and cellular internalization while simultaneously enabling the targeting of diverse cell populations within the tumor microenvironment or cells expressing multiple receptor types on their surface. One of the first papers describing the evaluation of the effectiveness of FA, vitamin B12, or BIO-functionalized polymeric materials was published in 2004. Russell-Jones et al. reported that the cells overexpressing the receptors for FA or vitamin B12 also overexpress receptors for BIO.^29^ To date, researchers have documented the development of both taxoid conjugates and doxorubicin-loaded NPs functionalized with the dual-targeting ligands biotin (BIO) and FA for enhanced cancer-specific delivery.^23,30^ The mixed monolayers were prepared with 1% FA or BIO on a long PEG linker. HeLa (human cervical cancer) and MCF7 (human breast adenocarcinoma) cells exhibited significantly higher targeting and attachment compared to nontumorigenic MCF10A breast epithelial cells.^31^ The multifunctional pH-responsive silica-coated NPs conjugated to BIO, FA, and doxorubicin were obtained for increasing tumor drug accumulation and endosomal drug release properties.^32^ Recently, the advantageous potential of FA- and BIO-functionalized liposomes was reported.^33^ Further progress can be obtained by the development of dual-targeted micelles for anticancer drug delivery. The preliminary study on the PLA–PEG micellar system dual-targeted with FA and BIO has shown some cytotoxic potential against OVCAR-3 cells.^34^ However, further study is needed to optimize micelles’ properties and to assess their pharmaceutical potential. There is also a need to conduct a more detailed study in vitro and in vivo, involving several cell lines differing in receptor characteristics to better understand the functionality of dual-targeted NPs. Therefore, the aim of the study was to analyze the influence of the type and amount of targeting ligand (FA and/or BIO) on micelles’ morphology, physicochemical properties, drug loading and release properties, cell internalization, and cytotoxicity against cells that differ in their surface receptor characteristics. For this purpose, three types of micelles were obtained: decorated with one type of ligand (FA or BIO) and dual-targeted with both ligands (FA and BIO). Moreover, each type of functionalized micelle was obtained in three options, differing in the amount of functionalized polymer (10, 25, or 50%). Dual-targeted micelles were also compared with a mixture of single-targeted micelles. To the best of our knowledge, this is the first study on dual-targeted micelles with FA and BIO that involves comprehensive characteristics and analysis of in vitro cytotoxic effect against a few types of cancer cells and two different cancer models in vivo.

## 2 Materials and Methods

### 2.1 Materials

Poly­(l,l-lactide)-*co*-poly­(ethylene glycol) (PLLA–PEG), poly­(l,l-lactide)-*co*-poly­(ethylene glycol)-folic acid (PLLA–PEG-FA), and poly­(L,l-lactide)-*co*-poly­(ethylene glycol)-biotin (PLLA–PEG-BIO) were obtained from RuixiBiotechCo. Ltd. (China). The number-average molar masses (*M*_n_) of PLA and PEG blocks were 2000 and 5000, respectively. Dtx was purchased from LC Laboratories (Woburn, MA, USA). All types of organic solvents were purchased from Merck (Poznań, Poland).

### 2.2 Cell Culture

All cells were obtained from the American Type Culture Collection (ATCC, Manassas, VA, USA). Human fibroblasts (WI-38; CCL-75) and mouse melanoma (B16-F10; CRL-6475) were used as cell line models that do not express receptors of folic acid (FAR) and SMVT that recognize BIO.^35,36^ The human cervical cancer cells (HeLa; CCL-2) and mice mammary cancer cells (4T1; CRL-2539) were used as cell culture models with high expression of FAR and SMVT.^35,37,38^ Human breast cancer cell line (MCF-7; HTB-22) and human breast adenocarcinoma (SK-BR-3; HTB-30) were the model cells with very low expression of FAR.^35,36^

WI-38, B16-F10, and HeLa cells were cultured in Dulbecco’s modified Eagle’s medium-high glucose (DMEM). MCF-7 cells were cultured in Eagle’s minimum essential medium and SK-BR-3 cells in McCoy’s Medium Modified. Each medium was supplemented with 10% fetal bovine serum, 100 U/mL penicillin, and 100 μg/mL streptomycin. The medium of MCF-7 cells was additionally supplemented with 0.01 mg/mL human recombinant insulin. The medium of experimental cell cultures was also supplemented with 10 mM *N*-(2-hydroxyethyl)­piperazine-*N*′-ethanesulfonic acid. The cells were maintained at 37 °C in a humidified atmosphere of 5% CO_2_.

### 2.3 Preparation of Micelles

The micelles were obtained by a cosolvent evaporation method according to the previously published procedure.^39^ Three kinds of polymers were used for preparation of micelles PLA–PEG, PLA–PEG-FA, and PLA–PEG-BIO, and the composition of particular formulations is presented in Table 1. The polymer or mixture of polymers (Table 1) was dissolved in chloroform (5% w/v), mixed with deionized water, and added to each vial under stirring. The incorporation of the drug (Dtx) or fluorescence agent (FITC) was performed by the addition of the drug dissolved in ethanol (20% w/v) or FITC in ethanol (1% w/v) to the micellar solution. Drug-free micelles were filtered through syringe filters (0.8 μm) and lyophilized. The drug-loaded micelles and FITC-loaded micelles were centrifuged at 3000 rpm for 5 min, and the supernatant was lyophilized. The lyophilized micelles were stored at 4 °C for further analysis. The lyophilized micelles have been reconstituted in aqueous or organic solution (depending of experiment) directly before analysis.

**Table 1:** Composition of the Obtained Single- and Dual-Targeted Polymer Micelles

micelles‘ name	PLA–PEG [% w/w]	PLA–PEG-FA [% w/w]	PLA–PEG-BIO [% w/w]
FA10	90.0	10.0	
FA25	75.0	25.0	
FA50	50.0	50.0	
BIO10	90.0		10.0
BIO25	75.0		25.0
BIO50	50.0		50.0
FABIO10	90.0	5.0	5.0
FABIO25	75.0	12.5	12.5
FABIO50	50.0	25.0	25.0

### 2.4 Characterization of Micelles

#### 2.4.1 Microscopic Evaluation

Cryo-TEM imaging was performed using a Tecnai F20 X TWIN microscope (Thermo Fisher Scientific, formerly FEI Company) with a field emission gun at 200 kV. Images were captured using a Gatan Rio 16 CMOS 4k camera and processed via the Gatan Microscopy Suite software (Gatan Inc., Pleasanton, CA, USA). Quantifoil R 2/2 holey carbon grids were plasma-treated for 15 s using a Femto plasma cleaner (Diener Electronic) with oxygen plasma. The preparation of the sample was conducted by vitrification of the aqueous solutions on grids with a holey carbon film (Quantifoil R 2/2; Quantifoil Micro Tools GmbH, Groβlöbichau, Germany). A 3 μL sample droplet was applied to the grid, blotted with filter paper, and rapidly frozen in liquid ethane using a Vitrobot Mark IV automated plunge freezer (Thermo Fisher Scientific). The vitrified specimens were stored in liquid nitrogen until analysis. Samples were transferred to a cryo-TEM holder (Gatan 626) and imaged at −178 °C.

#### 2.4.2 Zeta Potential

Dynamic light scattering (DLS) was used to measure the Zeta potential. The measurement was conducted at 25 °C with a 90° separation angle using a Malvern Zetasizer Nano spectrometer (Malvern Instrument, UK). Water solutions of micelles with a concentration of 1 mg/mL were tested after filtration through 0.8 μm syringe filters.

#### 2.4.3 Determination of the Amount of Surface Ligands

The quantity of ligands in the prepared micelles was determined by absorbance measurements using the standard curve method for FA and a colorimetric test to determine the BIO content.

##### 2.4.3.1 FA Measurements

Solutions of the tested micelles in dimethyl sulfoxide (DMSO) of 1 mg/mL were used for quantitative assessment. The multiplate reader (Spark, Tecan) was used for spectrophotometric measurements at a wavelength of 365 nm.

##### 2.4.3.2 Colorimetric Biotin Assay Kit

The quantitative analysis of BIO in the micelles was analyzed with the use of the Colorimetric Biotin Assay Kit (Sigma-Aldrich). The micelle solutions were prepared in deionized water at a concentration of 1 mg/mL filtered through 0.8 μm syringe filters. D-Biotin solution was used as a positive control, and deionized water was used as a negative control. Absorbance was measured using a multiplate reader (Spark, Tecan) at a wavelength of 500 nm. The BIO content was calculated according to the assay protocol.

#### 2.4.4 Measurements of the Contact Angle

Contact angle measurements were performed using a CAM101 goniometer (KSV Instruments), which was equipped with a 640 × 480 pixel resolution camera and an external temperature controller (Intelligent Digital Controller OMRON5EGN). The analysis was performed at room temperature. A drop of sample of micelles at a concentration of 1 mg/mL (2–4 μL) was placed with the use of a glass syringe on a coverslip. The results of contact angle measurement are the average of 30 photos taken at a speed of 1 photo/s in 3 different places on the coverslip. The CAM2008 program was used for the data analysis and calculations.

#### 2.4.5 NMR Analysis

The lyophilized micelles, biotin, and FA were analyzed by means of proton (1H NMR) nuclear magnetic resonance spectroscopy (Bruker, AVANCE II Ultrashiels Plus spectrometer; 600 MHz). The samples were dissolved in deuterated DMSO (DMSO-*d*_6_) prior to analysis. Chemical shifts (δ) are given in ppm.

### 2.5 Drug Loading and Release Study

The encapsulation efficiency (EE) of drugs and drug LC was calculated using the following equations (eqs 1 and 2)­

1 $$EE\;\%=\frac{weight\;of\;the\;drug\;in\;the\;micelles}{weight\;of\;the\;drug\;added\;to\;the\;micelles}\times 100\%$$

The LC was calculated using the following equation

2 $$LC\;\%=\frac{weight\;of\;loaded\;drug}{weight\;of\;polymeric\;micelles}\times 100\%$$

To determine the EE and LC, the lyophilized samples were dissolved in ethanol (1 mg/mL) and analyzed via high-performance liquid chromatography (HPLC).

The dialysis method was used for the analysis of the release of Dtx from the micelles. Lyophilized micelles (2 mg/mL in phosphate-buffered saline (PBS), pH 7.4) were loaded into dialysis tubes (Float-A-Lyzer G2, MWCO 3.5–5 kDa; Spectra Por). The tubes were immersed in 45 mL of PBS, which was regularly exchanged to maintain sink conditions. All micellar solutions remaining in the dialysis bag were collected at 1, 6, 24, 48, 72, and 168 h, lyophilized, and then redissolved in ethanol (1 mL) for HPLC analysis. The experiments were performed in triplicate.

A HPLC was used for the quantitative analysis of the drug (VWR/Hitachi). LiChrospher 100 RP-18 (5 μm) LiChroCART 250-4 and LiChrospher guard column 100 RP-18 (5 μm) LiChroCART 4–4 (Merck) were used as a stationary phase. Acetonitrile and water (60:40, v/v) were used as the mobile sample (flow rate of 1 mL/min). Dtx was detected at 227 nm by a diode array detector. The standard curve of the peak area versus drug concentration (0.025–500 μg/mL) was obtained by linear regression analysis (*R* ^2^ = 0.999).

### 2.6 Cell Internalization Study

The in vitro cell internalization of FITC-loaded micelles single-targeted (FA10 and BIO10) or dual-targeted (FABIO10) was studied on cells with the high expression of receptors of FA and BIO (HeLa and 4T1) and cells with low expression of FAR (WI-38 and B16-F10). Cells were cultured under standard conditions. Cells were seeded in 8-well Ibidi chambered coverslips at a density of 5 × 10^5^ cells per well in 300 μL of medium. After 24 h of incubation, the growth medium was replaced with 200 μL of suspended micelles at a concentration of 500 μg/mL and incubated for an additional hour. After 45 min, Hoechst 33342 dye was added (without removing micelles) and incubated for the last 15 min. Cells were then washed with Dulbecco’s PBS 3 times and observed under a confocal microscope Zeiss LSM710.

### 2.7 In Vitro Cytotoxicity Study

The in vitro cytotoxicity of drug-free micelles single-targeted with FA (FA10, FA25, and FA50), BIO (BIO10, BIO25, and BIO50), and dual-targeted with FA and BIO (FABIO10, FABIO25, and FABIO50) was conducted according to the ISO 10993-5 and ISO 10993-12 standard. The WI-38 cells were cultured in DMEM under standard conditions as described in Section 2.2. To assess cytotoxicity, 100 μL of cell suspension, containing 6 × 10^3^ cells, was seeded into each well of a 96-well plate and incubated for 24 h. Then, the medium was exchanged for the medium (200 μL) containing micelles at the concentrations of 20, 50, and 100 μg/mL. The micelles were prepared directly before the experiment by suspension in DMEM, filtering through 0.2 μm syringe filters, and diluting (20–100 μg/mL). The cells were exposed to the tested compounds for 72 h. Untreated cells were used as a negative control, and 5% DMSO-treated cells as the positive control. The viability of cells was evaluated with the use of the Sulforhodamine B (SRB) assay. After treatment, the media was removed, and cells were fixed with 10% trichloroacetic acid (4 °C), triple-rinsed with deionized water, and treated with SRB dye. Absorbance was measured at 570 and 690 nm (reference) using a Spark microplate reader (Tecan).

Cytotoxicity of Dtx-loaded micelles was studied against the cell culture with the high expression of receptors of FA and BIO (HeLa and 4T1) and cells with low expression of FAR (MCF-7 and SK-BR-3). For the viability experiment, cells were seeded in 96-well plates at a density per well of 3 × 10^3^ (HeLa and 4T1) or 6 × 10^3^ for SK-BR-3 and MCF-7 and cultured for 24 h to provide cell adhesion. After 24 h, the culture medium was replaced with fresh medium containing either the free drug or drug-loaded micelles. The free Dtx and Dtx in micelles (FA10, FA25, FA50, BIO10, BIO25, BIO50, FABIO10, FABIO25, and FABIO50) was analyzed at the concentration range of 0–640 nM (HeLa, SK-BR-3, and MCF-7) and 0–102.4 μM (4T1). Additionally, Dtx in a 1:1 (w/w) mixture of single-targeted micelles (FA10 + BIO10) was analyzed against HeLa and 4T1 cells. Free Dtx was dissolved in ethanol and diluted in the culture medium. Micelles were suspended in culture medium directly before the experiment and filtered through a 0.2 μm membrane filter. Untreated cells were used as negative control and 5% DMSO-treated cells as positive control. Cells were incubated with the test preparations for 72 h. Each sample was analyzed in at least 8 repetitions in experiment, and each experiment was conducted thrice. The viability of cells was determined by using the SRB assay.

### 2.8 Fluorescence Imaging Cytometer Analysis

For comparison of the effect of Dtx delivered in dual-targeted micelles (FABIO10) or a mixture of FA10 and BIO10 (FA10 + BIO10) on 4T1 cells, an additional study was conducted with the use of a fluorescence imaging cytometer. A series of cytometric analyses were conducted to evaluate the cell viability, redox homeostasis disorders, and the induction of cell apoptosis after administration of 1.28 μM Dtx encapsulated in FABIO10 or FA10 + BIO10 micelles. After 72 h of incubation with the analyzed formulations, the cells were detached by trypsinization, centrifuged, and counted. Based on the obtained culture count results, samples were prepared for individual cytometric analyses, in accordance with the manufacturer’s recommendations. All the cytometric analyses were performed using the NucleoCounter NC-3000 fluorescence imaging cytometer (ChemoMetec) and NucleoView NC-3000 software (version 2.1.25.12).

#### 2.8.1 Analysis of the Cell Number and Viability

Cytometric analysis of 4T1 cells viability was performed according to the Viability and Cell Count Assay protocol using DAPI (population of nonviable cells) and acridine orange (all cells detection) staining.^40^

#### 2.8.2 Annexin V Analysis

Analysis of the apoptosis process was performed by using Annexin V conjugated to a fluorescent dye (Annexin V-CF488A). Since the binding of Annexin V to phosphatidylserine may also occur in the absence of integrity of cell membranes in the late phase of apoptosis, propidium iodide (PI) is applied because it does not penetrate intact biological membranes and enables differentiation of subpopulations of early and late apoptotic cells. The study used samples prepared by suspending 3 × 10^5^ cells in 100 μL of Annexin V Binding Buffer. Then, 2 μL of Annexin V-CF488A conjugate and 2 μL of Solution 15 reagent (500 μg/mL Hoechst 33342) were added and incubated at 37 °C for 15 min using a heating block. In the next step, the cells were centrifuged (400*g*, 5 min) and washed twice with Annexin V Binding Buffer. The obtained cell pellets were resuspended in 100 μL of Annexin V Binding Buffer, to which 2 μL of Solution 16 reagent (500 μg/mL PI) was added. The samples were immediately placed on NC-Slides A2 slides (ChemoMetec) and analyzed according to the Annexin V Assay protocol.

#### 2.8.3 Mitochondrial Membrane Potential Analysis

The lipophilic, cationic dye JC-1 was used to study the mitochondrial membrane potential according to the procedure described previously.^40^ Immediately before the analysis, the cells were resuspended with 0.25 mL of Solution 8 reagent (1 μg/mL DAPI solution in PBS) to detect the late phase of apoptosis. The prepared samples were placed on eight-chamber NC-Slides A8 slides (ChemoMetec) and analyzed according to the Mitochondrial Potential Assay protocol.

#### 2.8.4 Cell Cycle and DNA Fragmentation Analysis

Samples for the analysis were prepared by suspending 1 × 10^6^ cells in 0.5 mL of PBS and fixed by adding 4.5 mL of 70% cold ethanol. After 24 h of incubation of the fixed cells at 0–4 °C, the samples were centrifuged and washed with 5 mL of PBS. The pellets were suspended in 0.5 mL of Solution 3 reagent containing DAPI (1 μg/mL) and 0.1% Triton X-100 in PBS (ChemoMetec). Stained samples were placed on NC-Slides A8 eight-cell slides and analyzed according to the Fixed Cell Cycle-DAPI Assay or DNA fragmentation Assay protocol.

#### 2.8.5 Analysis of Intracellular Levels of Reduced Thiols

The assay was performed using VitaBright-48 (VB-48) reagent following the protocol described previously.^41^ The stained cells were placed on 8-chamber NC-Slides A8 slides and analyzed using the Vitality protocol Assay according to the manufacturer’s instructions.

Two-way ANOVA followed by Tukey’s post hoc test was used to determine the significance.

### 2.9 In Vivo Analysis

Experiments were conducted on female BALB/c and C57BL/6 mice (8 to 10 week old) from the National Research Institute of Oncology (Gliwice, Poland). All animal experiments were conducted in accordance with the National Institutes of Health Guide for the Care and Use of Laboratory Animals and the 3R principles, with approval from the Local Ethics Commission for Animal Experiments in Katowice (permission 54/2023 of October 26, 2023). Mice were housed in a specific pathogen-free facility at the Maria Skłodowska-Curie National Research Institute of Oncology, Gliwice Branch (Poland). They were provided with a complete pathogen-free standard diet (Altromin International, Lage, Germany).

#### 2.9.1 Micelles Tissue Internalization

Murine breast cancer, 4T1, and melanoma, B16-F10, were injected subcutaneously (lower flank) with 2 × 10^5^ cells. Tumors were measured using calipers, and their volumes were calculated according to the formula: volume = width^2^ × length × 0.52. When the tumor volume reached 50 mm^3^, micelles with FITC were injected intravenously at a dose of 1 mg/mice in 200 μL of PBS. Mice in the control group received 200 μL of PBS. Micelles were administered 2 times in 2 days. 24 h after the second administration of micelles, tumors were collected and visualized using an IVIS XR In vivo imaging system (Caliper Life System, Germany). Radiance efficiency [(p/sec/cm^2^/sr)/(μW/cm^2^)] was set to the same range for all measurements (3 × 10^6^ to 2 × 10^7^ for 4T1 and B16F10 tumors).

#### 2.9.2 Therapy of Murine Breast Cancer Tumors with Micelles

Murine breast cancer (4T1) and melanoma (B16-F10) were injected subcutaneously (lower flank) with 2 × 10^5^ cells. When the tumor volume reached about 50 mm^3^, micelles with Dtx were injected intravenously at a dose of 200 μg/mice in 200 μL of PBS. The following groups of mice received 200 μL of: (1) PBS, (2) free Dtx, (3) Dtx-loaded micelles with no ligand, (4) Dtx-loaded FA10 micelles, (5) Dtx-loaded BIO10 micelles, (6) Dtx-loaded FABIO10 micelles, and (7) Dtx-loaded FA10 + BIO10 micelles. In all groups that received Dtx its concentration was 10 mg/kg. Micelles were administered 3 times every other day on days 7, 9, and 11 in the breast cancer model and 11, 13, and 15 in the melanoma model. Tumors were measured with calipers; tumor volumes were determined using the formula: volume = width^2^ × length × 0.52.

### 2.10 Statistical Analysis

The multiple-group comparisons were performed using one-way ANOVA. The normality of the distribution was verified by the Shapiro–Wilk test. The homogeneity of variance was checked using Levene’s test. The multiple-group comparisons were performed using one-way ANOVA with the posthoc Tukey test or the Kruskal–Wallis with the multiple comparison posthoc test depending on the variable distribution and variance homogeneity.

Statistical analysis was performed using Statistica 13 software. All the results are expressed as means ± SD or means ± SEM. All the results are expressed as means ± SD. The *P* value of <0.05 was considered statistically significant.

## 3 Results

### 3.1 Characteristics of Micelles

Three kinds of micelles were obtained from PLA–PEG, PLA–PEG-FA, and PLA–PEG-BIO (Table 1). Moreover, the single- and dual-targeted micelles were obtained with various contents of polymer functionalized with FA and/or BIO (10, 25, and 50% w/w) to analyze the influence of ligand amount on the properties of micelles. The morphology of all kinds of micelles was observed by means of cryo-TEM. As shown in Figure 1, the morphology of micelles was similar regardless of their composition. Also, the morphology of drug-free (Figure S1 A) and Dtx-loaded (Figure S1 B) micelles without the targeting moiety does not differ from single- and dual-targeted micelles (Figure 1). All kinds of micelles formed structures of dual morphology—spherical of ≈20 nm diameter and elongated shapes of 100–300 nm in length.

**Figure 1:**
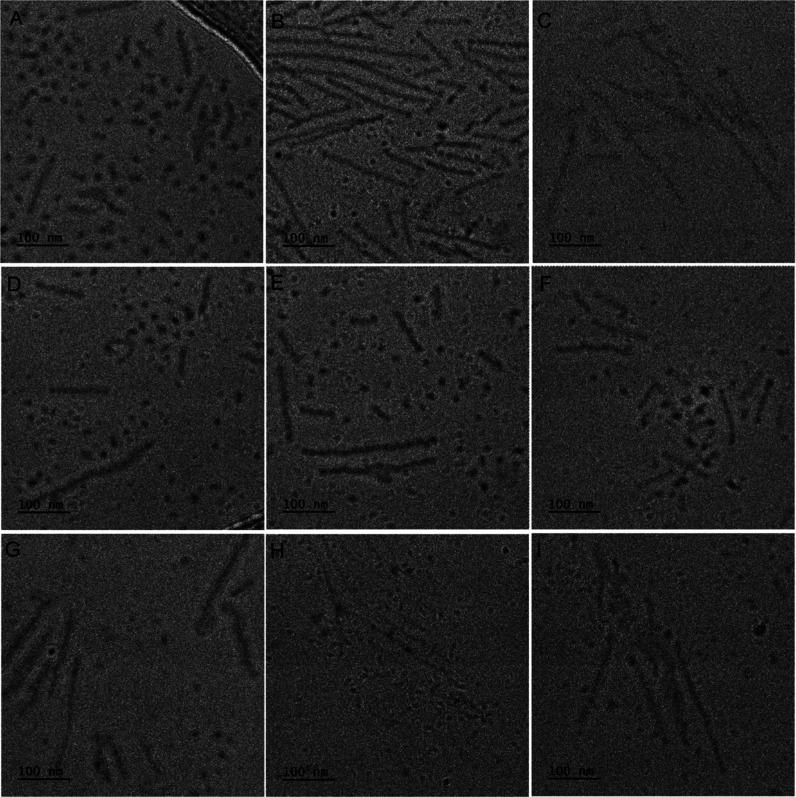
Cryo-TEM images of various kinds of micelles: FA10 (A), FA25 (B), FA50 (C), BIO10 (D), BIO25 (E), BIO50 (F), FABIO10 (G), FABIO25 (H), and FABIO50 (I).

The micelles were also characterized in terms of the content of surface ligands (FA and BIO) (Table 2). The amount of FA increased from 2.3 to 13.6 and 30.2 μg/mg in FA10, FA25, and FA50 micelles, respectively. In dual-targeted micelles, the FA content was lower due to the smaller amount of the single-functionalized polymer (PLA–PEG-FA) used for micelles’ formation, as shown in Table 1. Significantly smaller amounts of BIO were determined for both single- and dual-targeted micelles; however, in both cases, the amount of BIO gradually increased in BIO10, BIO25, and BIO50 (and FABIO equivalents) micelles. The comparison of the NMR spectra of FA, BIO, PLLA–PEG micelles, and single- and dual-targeted micelles is presented in Figures S2–S4. All kinds of the micelles characterized negative zeta potential, which was the highest for BIO50 (−4.7 mV) and the lowest for FA10 (−12.4 mV). The increase of ligand content correlated with the increase of zeta potential. The contact angle of all kinds of micelles was smaller than the contact angle of pure water. The highest contact angle was determined for micelles functionalized with BIO (53°–54°) and the lowest for FA10 and FABIO50.

**Table 2:** Physicochemical Characterization of the Micelles (Mean ± SD, *n* = 3)

kind of micelles	folate content [μg/mg of liophilized micelles]	biotin content [μg/mg of liophilized micelles]	potential zeta (ζ) [mV]	contact angle (θ) [°]^a^
**FA10**	3.8 ± 0.8		–12.4 ± 0.1	42.0 ± 1
**FA25**	13.6 ± 0.3		–10.2 ± 0.5	46.0 ± 3
**FA50**	30.2 ± 1.0		–10.0 ± 0.1	51.0 ± 1
**BIO10**		0.61 ± 0.2	–10.8 ± 0.1	53.0 ± 4
**BIO25**		1.94 ± 0.1	–9.7 ± 0.4	54.0 ± 1
**BIO50**		5.28 ± 0.5	–4.7 ± 0.3	53.0 ± 2
**FABIO10**	0.1 ± 0.0	0.38 ± 0.1	–11.0 ± 0.6	50.0 ± 1
**FABIO25**	5.4 ± 0.1	1.41 ± 0.1	–9.1 ± 0.4	51.0 ± 2
**FABIO50**	14.2 ± 0.3	3.19 ± 0.2	–7.8 ± 0.5	43.0 ± 1

a The contact angle of water was 55.0 ± 3.0.

### 3.2 Drug Loading Properties of PLA–PEG Micelles

The drug loading and release properties were analyzed for all kinds of micelles. Table 3 presents a comparison of the EE and LC of Dtx. All kinds of single- and dual-functionalized micelles provided high Dtx encapsulation capacity. Also, the increase of ligand content influenced the increase of EE and LC. The highest Dtx loading capacity was shown by BIO50 (EE = 71.2%), and slightly lower Dtx loading capacity was observed in FABIO50 (EE = 67.31%) and FA50 (EE = 66.65%).

**Table 3:** Dtx EE and LC in Various Kinds of Micelles (Mean ± SD, *n* = 3)

micelles‘ name	EE [%]	LC [%]
FA10	52.27 ± 4.35	8.71 ± 0.73
FA25	50.40 ± 2.92	8.40 ± 0.49
FA50	66.65 ± 1.53	11.10 ± 0.26
BIO10	45.85 ± 10.19	7.64 ± 1.70
BIO25	51.43 ± 6.73	8.57 ± 1.12
BIO50	71.20 ± 2.80	11.86 ± 0.47
FABIO10	49.40 ± 2.54	8.23 ± 0.42
FABIO25	57.18 ± 4.72	9.53 ± 0.79
FABIO50	67.31 ± 2.70	11.21 ± 0.45

The regular release profile of Dtx was observed for single- and dual-targeted micelles, as presented in Figure 2. Moreover, the process proceeded similarly for all kinds of micelles, and the amount of released Dtx exceeded 40% after 1 h and was beyond 48% after 6 h. The Dtx release was completed after 168 h.

**Figure 2:**
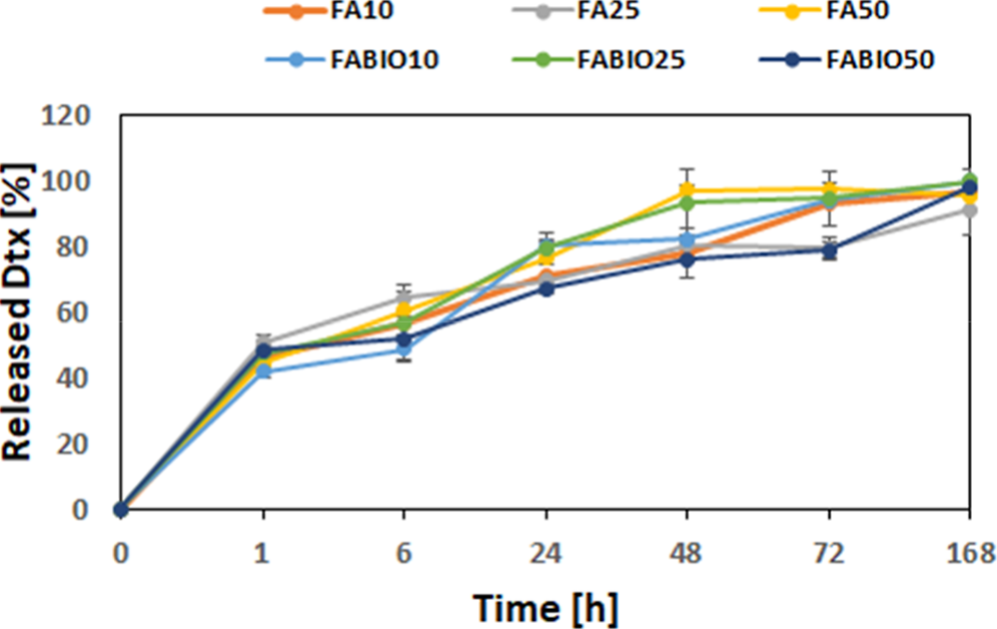
Comparison of the in vitro cumulative release profile of Dtx from micelles functionalized with FA (FA10, FA25, and FA50) and micelles dual-functionalized with FA and BIO (FABIO10, FABIO25, and FABIO50). Data shown as a mean ± SD (*n* = 3).

### 3.3 Cell Internalization of Micelles

Microscopic analysis was conducted for selected micellar formulations (micelles without ligand, FA10, BIO10, and FABIO10) to assess the cellular penetration of fluorescently labeled micelles containing FITC dye. Comparison of the fluorescence emission value at 520 nm for various kinds of FITC-loaded micelles has been presented in Figure S5. The results (Figure 3) demonstrated an increased uptake of micelles in the FAR- and SMTV-positive cell lines: 4T1 (mouse breast cancer) and HeLa (human cervical cancer) compared to significantly reduced penetration in the FAR- and SMTV-negative cell lines: B16-F10 (mouse melanoma) and WI-38 (human fibroblast). Notably, the micelles designed with dual ligands (FABIO10) targeting FA and BIO receptors exhibited considerably weaker penetration across all tested cell lines compared to their single-ligand counterparts.

**Figure 3:**
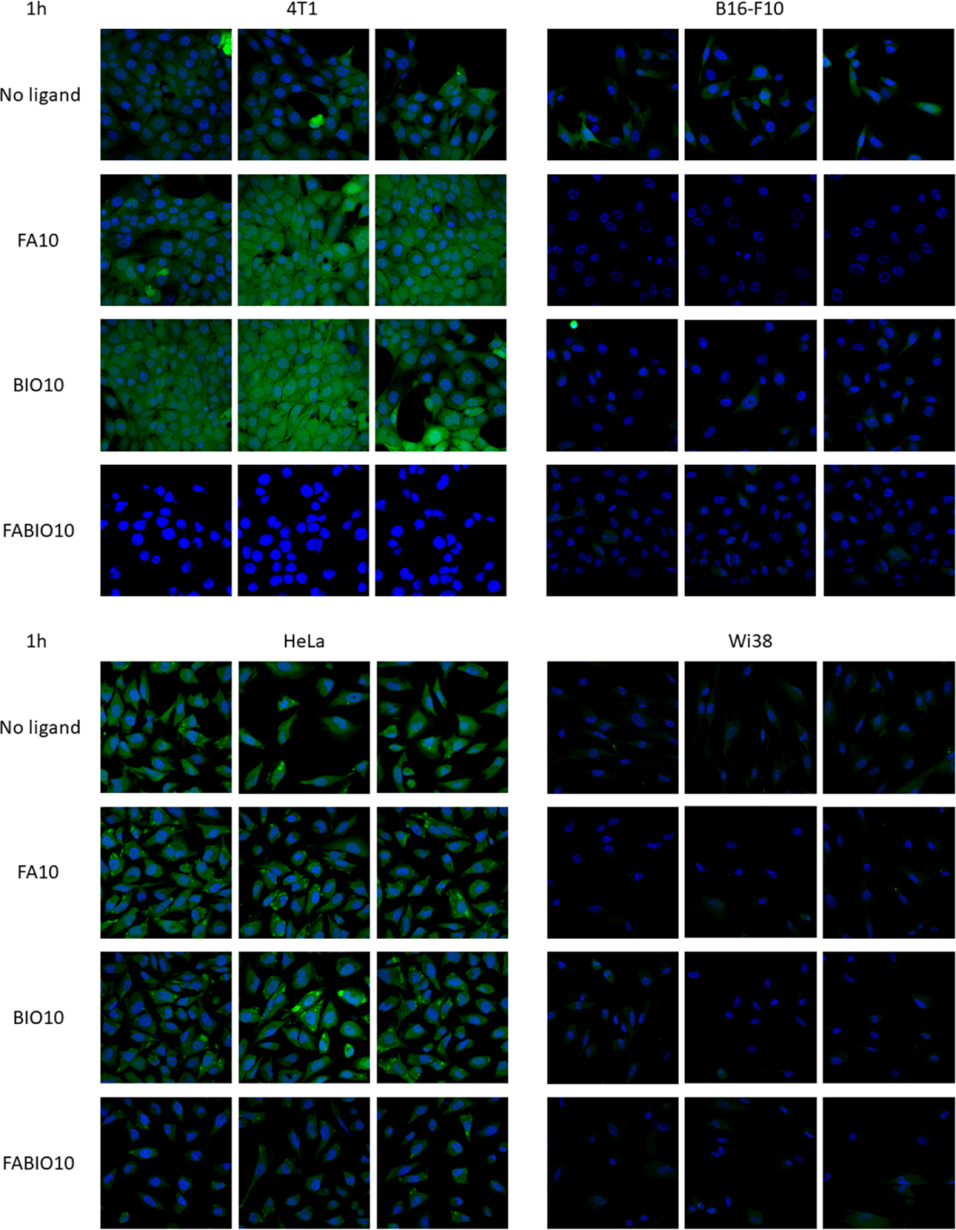
Microscopic observations of the in vitro internalization of micelles with the fluorescent FITC dye nonfunctionalized, functionalized with folic acid (FA10), biotin (BIO10), and dual-functionalized with FA and BIO (FABIO10) by 4T1, B16-F10, HeLa, and WI-38 cells. Nucleus stained with Hoechst 33342. Images were taken using a confocal microscope with a 20× objective magnification.

### 3.4 In Vitro Cytotoxicity Study

#### 3.4.1 Cytocompatibility of Drug-Free Micelles

Analysis of the in vitro cytocompatibility of all kinds of drug-free micelles was conducted according to the ISO 10993-5 and ISO10993-12 standards. The human fibroblasts (WI-38) were cultured in medium containing 20, 50, and 100 μg/mL of drug-free micelles, and the selected concentrations correspond to the highest concentration of drug-loaded micelles used in the cytotoxicity study. As presented in Figure 4, none of the analyzed micelles indicated cytotoxicity to the fibroblasts. The cells cultured in standard conditions did not differ from cells cultured in the presence of various kinds of drug-free micelles, regardless of concentration.

**Figure 4:**
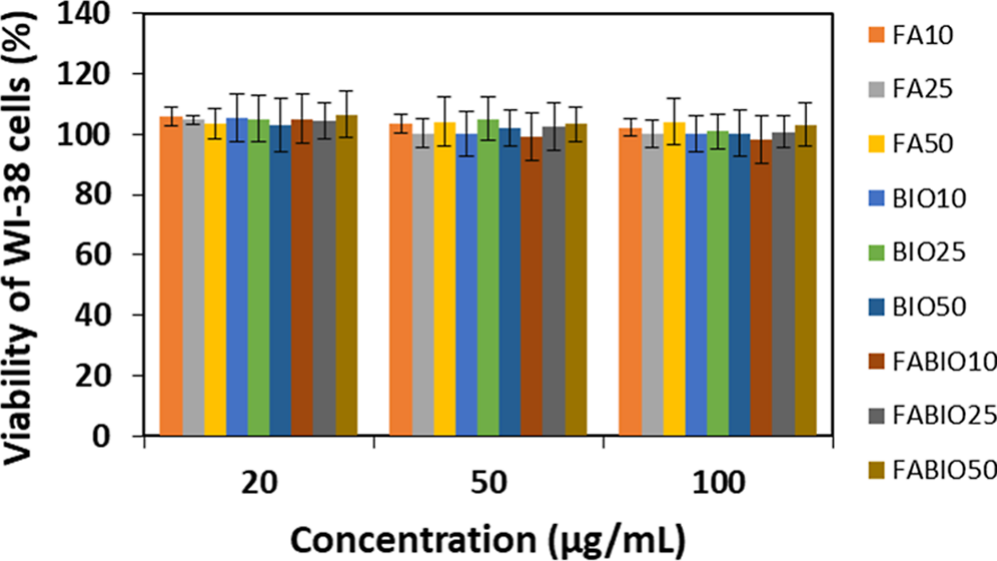
Viability of WI-38 cells in the presence of various types of drug-free micellar formulations: micelles functionalized with folic acid (FA10, FA25, and FA50), micelles functionalized with biotin (BIO10, BIO25, and BIO50), and micelles dual-functionalized with FA and BIO (FABIO10, FABIO25, and FABIO50). The results of the SRB assay are shown as mean ± SD; *p* < 0.05 compared with the negative control.

#### 3.4.2 Cytotoxicity of Dtx-Loaded Micelles against Cancer Cells with the Low Expression of FAR

Two cancer cell lines (SK-BR-3 and MCF-7) that characterize low expression of FAR and high expression of receptors for BIO were selected for comparison of the cytotoxic effect of Dtx delivered as a native drug or encapsulated in various kinds of micelles (Figure 5). The comparison of IC50 values has been presented in Table S1.

**Figure 5:**
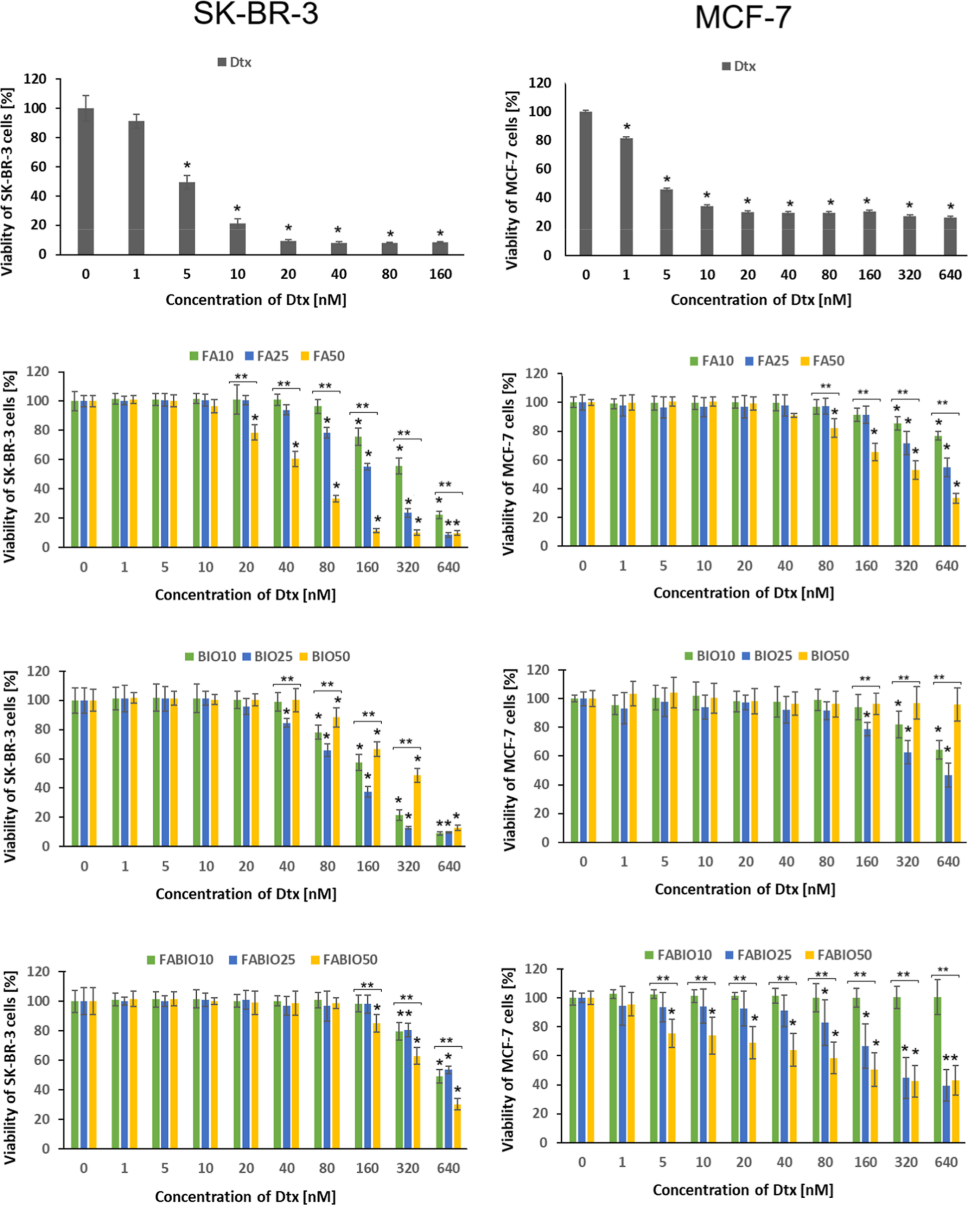
Viability of SK-BR-3 cells (left row) and MCF-7 cells (right row) in the presence of various formulations of Dtx: from the top, free drug (Dtx), Dtx in micelles functionalized with folic acid (FA10, FA25, and FA50), Dtx in micelles functionalized with biotin (BIO10, BIO25, and BIO50), and Dtx in micelles dual-functionalized with FA and biotin (FABIO10, FABIO25, and FABIO50). The results of the SRB assay are shown as mean ± SD; *p < 0.05 compared with negative control; **p < 0.05 compared between groups with various ligand contents.

The native Dtx exhibited a cytotoxic effect against SK-BR-3 at the concentration range of 5–160 nM. Dtx delivered in FA50 exhibited a significantly stronger cytotoxic effect (from 20 nM) compared to FA10 and FA25. Dtx in FA25 decreased cells’ viability from 80 nM and Dtx in FA10 at 160 nM. Also, the influence of BIO content in micelles on the cytotoxic effect was observed because Dtx in BIO25 decreased the growth of SK-BR-3 from 40 nM while Dtx in BIO10 and BIO50 from 80 nM. The lowest effectiveness was observed for Dtx in FABIO because they showed cytotoxic effect from 160 nM (FABIO50) or even from 320 nM (FABIO10 and FABIO25).

The MCF-7 cells showed decreased viability in the presence of native Dtx at the concentration of 1–640 nM. In the case of Dtx added in micelles decorated with FA, the decrease of MCF-7 cells’ growth was observed from 80 nM (FA50) or from 320 nM (FA10 and FA25). The effect of Dtx in micelles functionalized with BIO was similar to that observed for SK-BR-3 cells, so the Dtx in BIO25 showed the strongest cytotoxic potential (from 160 nM) in comparison to BIO10 (from 320 nM) and BIO50, which did not affect the cells’ growth even at 640 nM. Also, the cytotoxic effect of Dtx in FABIO50 micelles was significantly stronger (from 5 nM) compared to the Dtx in FABIO25 (from 80 nM) and in FABIO10, which did not show any effect at the whole studied concentration range. The results are consistent with the IC50 values presented in Table S1.

#### 3.4.3 Cytotoxicity of Dtx-Loaded Micelles against Cancer Cells with High Expression of Receptors for FA and BIO

In the next step, the cytotoxicity of all types of Dtx-loaded micelles was analyzed against cell lines with high expression of FA and BIO receptors (HeLa and 4T1). Viability of the HeLa cells exposed to the native Dtx and Dtx-loaded micelles is presented in Figure 6.

**Figure 6:**
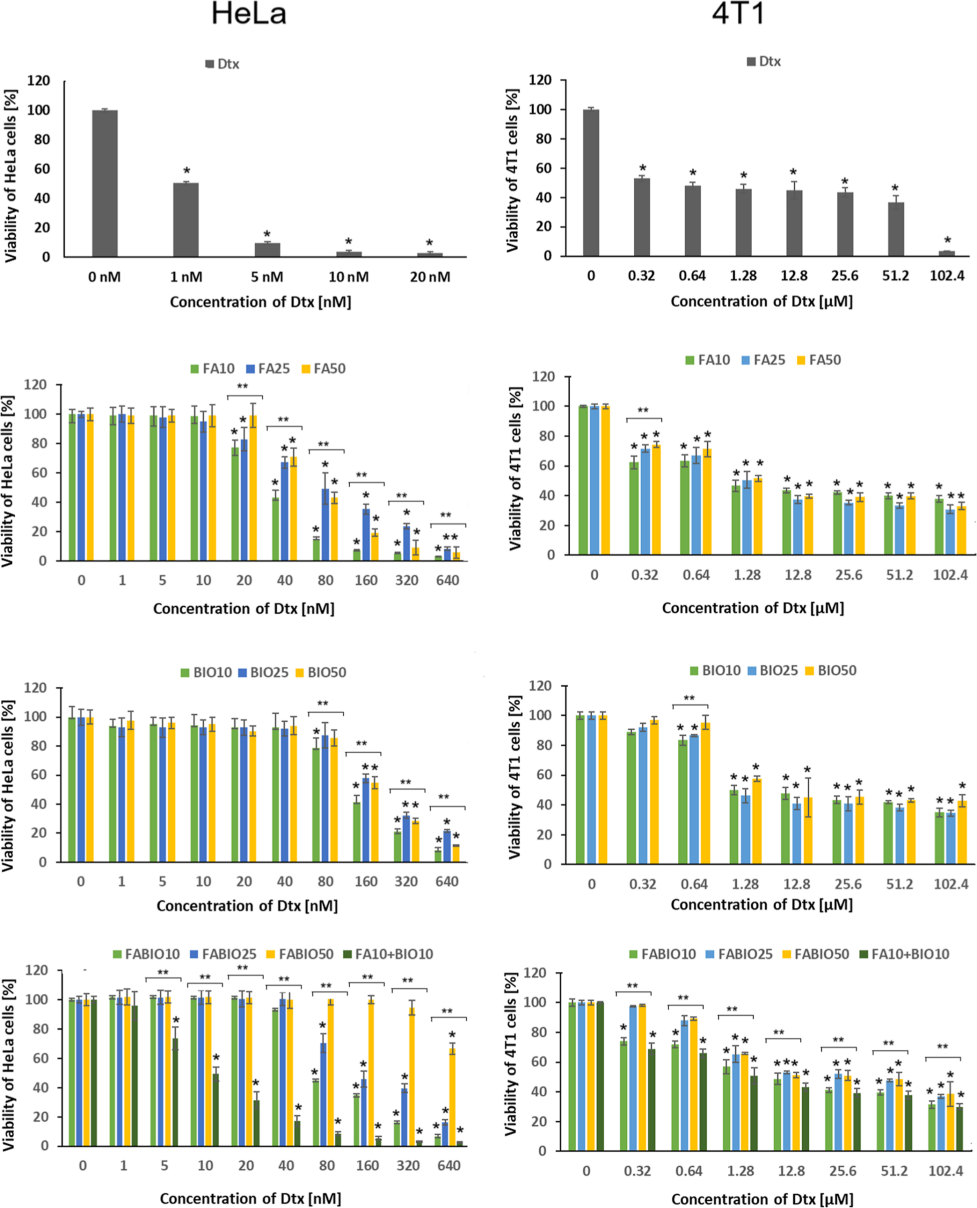
Viability of HeLa cells (left row) and 4T1 cells (right row) in the presence of various formulations of Dtx: from the top—free drug (Dtx), Dtx in micelles functionalized with folic acid (FA10, FA25, and FA50), Dtx in micelles functionalized with biotin (BIO10, BIO25, and BIO50), and Dtx in micelles dual-functionalized with FA and biotin (FABIO10, FABIO25, FABIO50, and FA10 + BIO10). The results of the SRB assay are shown as mean ± SD; *p < 0.05 compared with the negative control; **p < 0.05 compared between groups with various ligand contents.

The native Dtx decreased viability of HeLa cells already at the concentration of 1 nM. The single- and dual-functionalized micelles exhibited cytotoxic effects at higher concentrations. Moreover, the cytotoxic effect was dependent on both the type of ligand and its content. The Dtx in FA-functionalized micelles decreased cell viability from 20 nM (FA10 and FA25) or 40 nM (FA50). However, the most significant cytotoxic effect was observed for Dtx in FA10. The Dtx in FA25 and FA50 did not show significant differences at the range of 0–80 nM, but beyond this concentration, stronger cytotoxic effect was exhibited by Dtx in FA50 compared to FA25. A similar effect was observed for BIO-functionalized micelles, although no cytotoxic effect was observed at the concentration of 80 nM for Dtx in BIO10 or even 160 nM in the case of Dtx in BIO25 and BIO50. All kinds of FABIO micelles did not affect the HeLa cells’ viability at the range of Dtx concentration of 0–40 nM. Dtx in FABIO10 and FABIO25 decreased cells’ growth from 80 nM and FABIO50 at 640 nM. However, a significant difference was observed for Dtx added in the mixture of single-functionalized micelles (FA10 + BIO10) that caused a decrease in the growth of HeLa cells at 5 nM. The Dtx loaded in FA10 + BIO10 micelles also showed the lowest IC50 value (10 nM), as presented in Table S1.

The cytotoxic effect of native Dtx and Dtx-loaded micelles against 4T1 cells was studied at the concentration range of 0.32 μM–102.4 μM. All kinds of FA-functionalized micelles caused the decrease of 4T1 cell growth from 0.32 μM, and no significant differences between micelles with various densities of FA were observed. Significantly lower cytotoxic effects were exhibited by BIO-functionalized micelles. Dtx delivered in BIO10 and BIO25 decreased cell viability from 0.64 μM and BIO50 from 1.28 μM. Comparison of Dtx delivered in dual-targeted micelles (FABIO10) and in the FA10 + BIO10 mixture revealed a stronger cytotoxic effect at the lowest concentration (0.32 and 0.64 μM) compared to FA25 and FA50 micelles.

#### 3.4.4 Fluorescence Imaging Cytometer Analysis

For a more detailed analysis of the effect of Dtx in a mixture of single-targeted micelles (FA10 + BIO10) and dual-targeted micelles (FABIO10), the cells were studied by means of a fluorescence imaging cytometer. In the first part of the experimental panel, the viability of the mouse breast cancer cells (4T1) was assessed. The use of 1.28 μM Dtx in FABIO10 and a mixture of FA10 + BIO10 micelles affects the cell viability, with the percentages of DAPI-positive cells increasing from about 1% (control) to 22%, respectively. These results were reflected in microscopic images of 4T1 cell cultures (Figure 7A). The photographs illustrate the morphological changes observed in cells following exposure to micellar formulations of Dtx. In cells treated with both types of formulations, loss of intracellular contact and an increase in the number of spherical cells were observed when compared to the control. In order to evaluate the effect of Dtx-encapsulated micelles on the mitochondrial transmembrane potential, cell staining with JC-1 dye was performed using a NucleoCounter NC-3000TM fluorescence image cytometer. Disturbance of the mitochondrial transmembrane potential is associated with the early state of apoptosis. In turn, DAPI staining allows for the detection of cells in the late phase of apoptosis. Based on the results (Figure 7B), it was found that the use of Dtx in FABIO10 and a mixture of FA10 + BIO10 micelles resulted in an increase in the number of depolarized cells from about 23% (control) to 59%, respectively. Simultaneously, it was observed that the number of cells in the late-phase of apoptosis increased from 12% to 20% in the case of both formulations.

**Figure 7:**
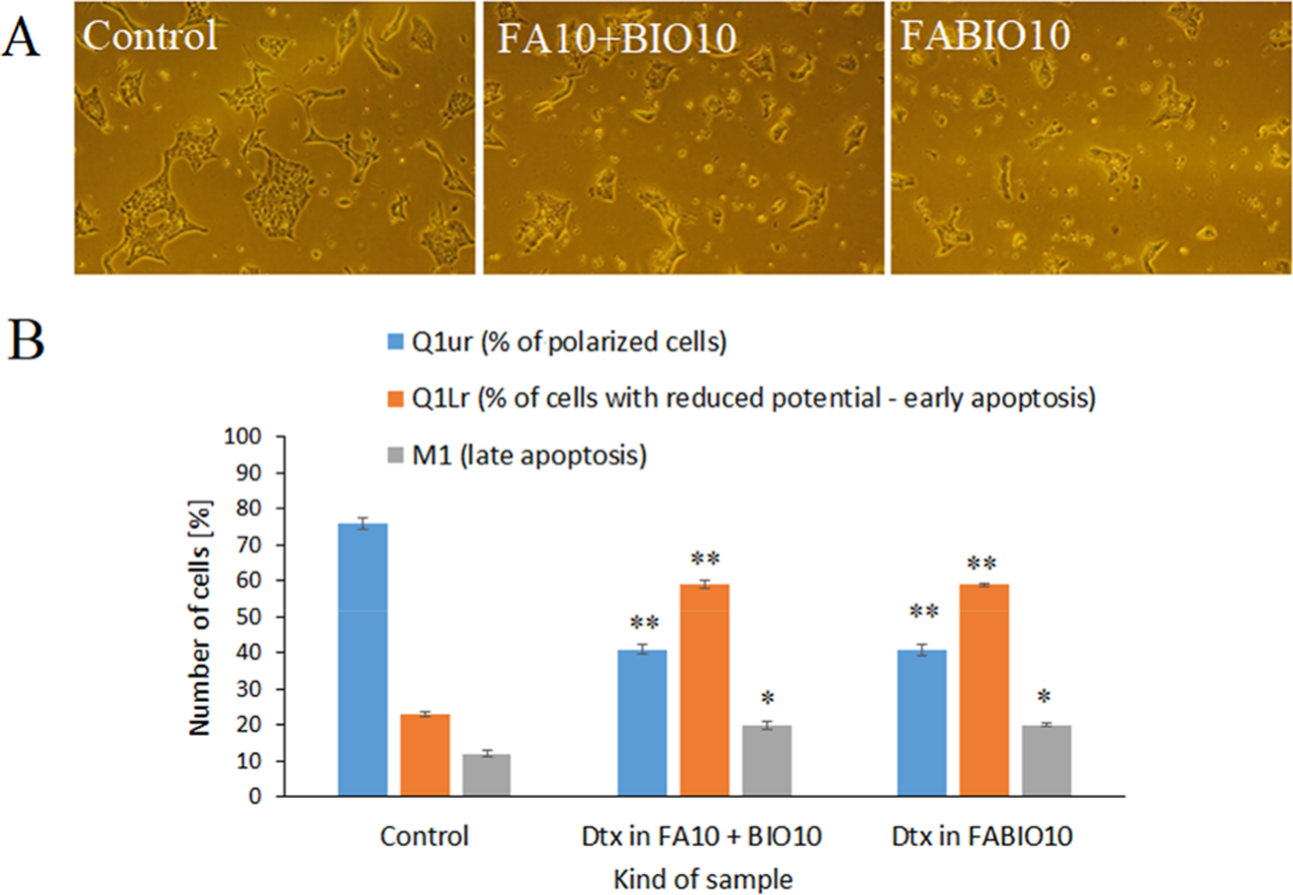
Effect of 1.28 μM of Dtx encapsulated in FABIO10 or a mixture of FA10 + BIO10 micelles on the morphology (A) and mitochondrial transmembrane potential (B) of 4T1 cells. Morphological changes in 4T1 cells were observed under the light inverted microscope at 40× magnification. The bar graph presents changes in the proportion of cells with polarized mitochondria and late-apoptotic 4T1 cells. Data are expressed as % of the controls; mean values ± SD (*n* = 9), *p < 0.5, and **p < 0.05.

During the analysis, individual phases of the cell cycle (subG1, G0/G1, S, and G2/M) were determined based on the quantitative DNA measurements. The obtained data indicate a significant change in the cell cycle profile after exposure to Dtx-loaded FABIO10 and FA10 + BIO10 micelles (Figure 8A). An increase in cells in the subG1 phase of the cell cycle was observed both in cells treated with Dtx in FA10 + BIO10 and FABIO10, from about 8% (control) to 52% and 55%, respectively. Simultaneously, a decrease in the number of cells in the G0/G1 phase from about 55% (control) to 15% and 18% and a decrease in the number of cells in the S phase from about 23% to 10% and 11% were noticed, respectively. Interestingly, Dtx encapsulated in FA10 + BIO10 micelles caused G2/M phase arrest with the increase of percentages of cells from about 13% (control) to 19%.

**Figure 8:**
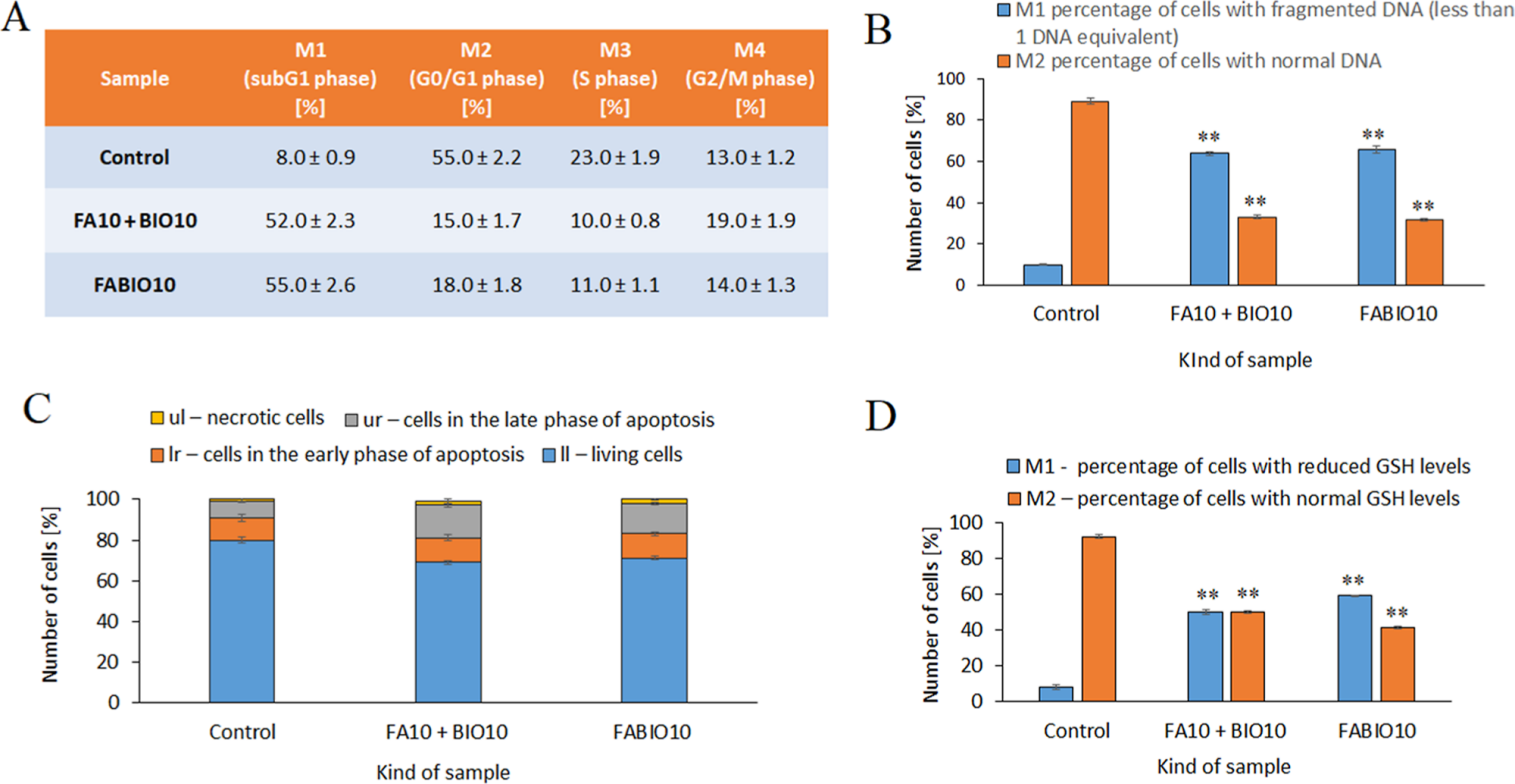
Cytometric analysis of cell cycle distribution in 4T1 cells treated with 1.28 μM of Dtx in FABIO10 or a mixture of FA10 + BIO10 micelles: cell cycle phases (A), DNA fragmentation (B), changes in Annexin V/PI intensity (C), and cellular GSH (D). Data are expressed as mean values ± SD, **p < 0.05.

DNA fragmentation assessment was performed using the fluorescent DAPI dye to measure intracellular DNA content. As a result of the experiment, it was found that the number of cells with fragmented DNA increased from about 10% (control) to 64% in the case of cells treated with Dtx in FA10 + BIO10 and 66% in the case of cells treated with Dtx in FABIO10 micelles (Figure 8B).

To detect programmed cell death, the Annexin V assay was conducted. One of the features of apoptosis is the translocation of phosphatidylserine to the outer surface of the cell membrane. The analysis showed an increase in the subpopulation of apoptotic 4T1 cells (Annexin V positive cells), in both the early and the late phases of apoptosis, with the percentages increasing from about 19% (control) to 28% (cells treated with Dtx loaded FA10 + BIO10 micelles) and 27% (cells treated with FABIO10 micelles) (Figure 8C).

Reduced thiol (GSH) levels were assessed using VitaBright-48 TM staining, which reacts inside the cells with the sulfhydryl moieties of thiols such as GSH. The reduction in GSH concentration in cells is associated with the progression of apoptotic processes and may consist of one of the causes of induction of this process. A significant increase in the number of cells with reduced GSH level was observed, from about 8% (control) to 50% in the case of cells treated with Dtx in FA10 + BIO10 and to 59% in the case of cells treated with Dtx in FABIO10 micelles (Figure 8D).

### 3.5 In Vivo Analysis of Tissue Internalization

The specificity of FITC fluorescent dye release in tumors was assessed in the 4T1 mouse breast cancer model (FA and BIO receptors are positive) and the B16-F10 mouse melanoma model (FA and BIO receptors are negative). After a single intravenous administration of different micelles, the fluorescence intensity of the tumor was assessed. It was shown that in the B16-F10 melanoma, no differences in micelle penetration into the tumor were observed (Figure 9A). In the breast cancer model, however, increased accumulation of FA10, BIO10, and FA10 + BIO10 micelles was observed (Figure 9B). In the case of dual-targeted micelles (FABIO10), the specific accumulation of micelles was at the level of micelles without a ligand.

**Figure 9:**
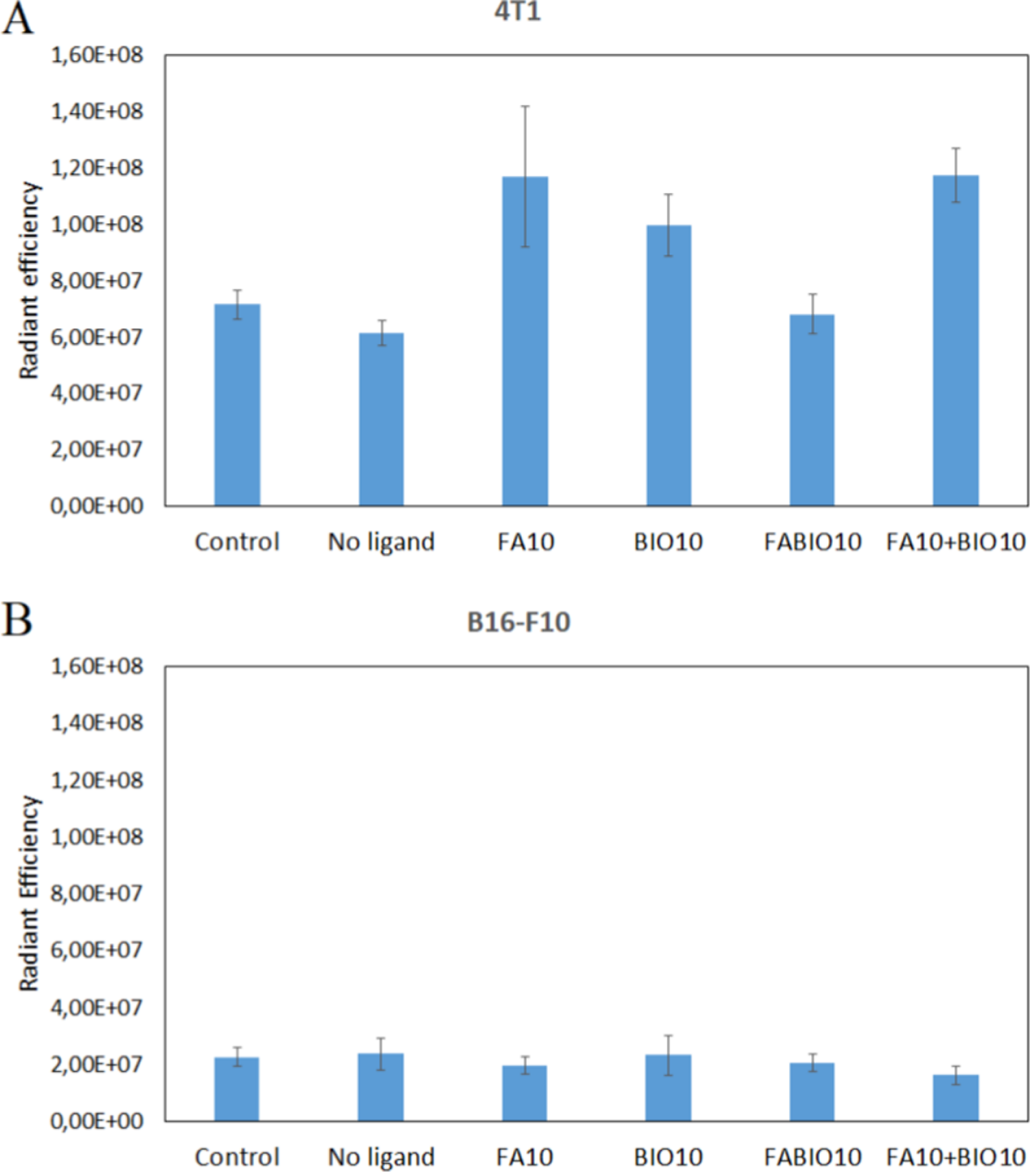
Internalization of different micelles (without ligand, FA10, BIO10, FABIO10, FA10 + BIO10) with FITC in tumor tissue-4T1 breast cancer (A), B16-F10 melanoma (B). Fluorescence of collected tissues was visualized using IVIS, indicated as radiant efficiency. The results are the mean value of 5 in each group and are shown as means ± SD.

### 3.6 Therapy of Breast Cancer-Bearing Mice with Dtx-Loaded Micelles

Finally, the therapeutic efficacy of the constructed Dtx-loaded micelles was verified in a mouse model of 4T1 breast cancer and B16-F10 melanoma. Mice with well-developed tumors were injected intravenously 3 times with different Dtx-loaded micelles. Tumor volumes were measured on each day. The statistically significant results were observed for FA10 and FA10 + BIO10 micelles compared to the control group in the 4T1 breast cancer model, which was characterized by high expression of FA and BIO receptors (Figure 10A). The tumor growth inhibition efficacy of dual-target micelles (FABIO10) was not greater than that of free Dtx, Dtx in BIO10, or in micelles without ligands. The Dtx in FA10 micelles inhibited tumor growth by 58% whereas Dtx in FA10 + BIO10 by 65%. In the case of Dtx-loaded BIO10 micelles, FABIO10, and micelles without the ligand, tumor growth inhibition was 40%, 32%, and 37%, respectively. Free Dtx inhibited tumor growth by 35%. In B16-F10 melanoma tumors, which were characterized by a low expression of both receptors, no differences in the tumor growth inhibition were observed (Figure 10B).

**Figure 10:**
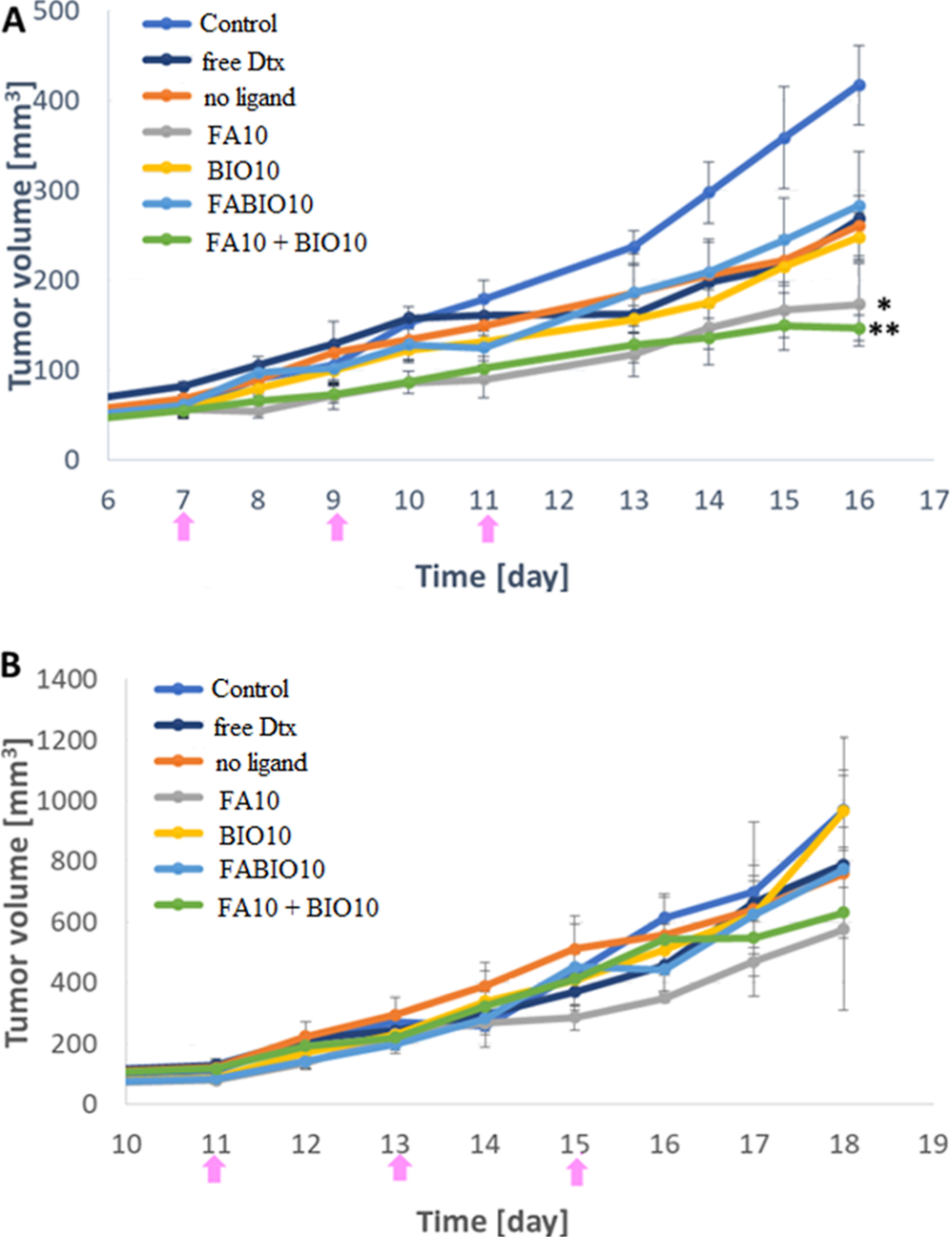
Inhibition of tumor growth after administration of the tested Dtx-loaded micelles (without ligands, FA10, BIO10, FABIO10, FA10 + BIO10) and free Dtx. Each formulation was administered 3 times every other day to mice bearing well-developed tumors, as indicated by pink arrows. All mice were administered docetaxel at a dose of 10 mg/kg body weight. Experiments were performed on 4T1 mammary carcinoma (A) and B16-F10 melanoma (B). Each group consisted of 5 mice. The tumor volume is shown as mean + SEM; *p < 0.05, **p < 0.01.

## 4 Discussion

Dual-targeted nanocarriers have been developed as a potential strategy to address tumor heterogeneity, which can arise between patients and between primary tumors and metastases, as well as within individual tumors.^14^ There is also a growing interest in the decoration of NPs with a combination of FA and BIO as targeting ligands.^31–35,42,43^ Although some preliminary study has been conducted to develop dual-targeted micelles for delivery of Dtx,^34^ a more detailed analysis is needed to discover their pharmaceutical potential. Therefore, the aim of the study was to analyze the influence of the type and amount of targeting ligand (FA and/or BIO) on their morphology, physicochemical properties, drug loading and release properties, cell internalization, and cytotoxicity against cells that differ in their surface receptor characteristics.

Three types of micelles were obtained: those decorated with one type of ligand (FA or BIO) and micelles dual-targeted with FA and BIO. Each type of functionalized micelle was obtained in three options, differing in the amount of functionalized polymer (10, 25, or 50% w/w) (Table 1) to analyze if the ligand content (density) affects micelles’ properties. The presence of ligand was verified, and the final content of FA and BIO is presented in Table 2. Analysis of micelles’ morphology did not show any difference regardless of polymeric composition, type of ligand, and its density (Figure 1). All kinds of materials formed structures of dual morphology—spherical (≈20 nm diameter) and elongated (≈100–300 nm length). Recent studies demonstrate that 180 nm filomicelles exhibit superior tumor tissue penetration and enhanced cellular uptake efficiency compared to both spherical micelles and longer 2.5 μm filomicelles.^44^

However, the differences in other parameters, such as contact angle and zeta potential, were observed between various kinds of micelles. The surface characteristics of NPs have an impact on the stability after intravenous injection as well as the interaction with mucus and epithelia.^45^ DLS was used for determination of zeta potential, which is the measurement of the surface charge of micelles, which influences their electrostatic interactions and affects the diffusion and stability of particles in solution.^46^

According to the effective drug delivery principles, optimal NPs should possess a zeta potential that is neutral or moderately negative.^47^ All kinds of micelles fulfill this requirement because they are characterized by negative zeta potential, although some differences were observed, and the highest value was determined for BIO50 and the lowest for FA10 (Table 2). Generally, the increase of ligand content correlated with the increase of zeta potential. The surface properties may also be characterized by the contact angle, which defines the spreading or wetting of a solid surface by a liquid. A low contact angle indicates that the liquid spreads well across the surface, demonstrating strong wettability of the solid.^48^ The contact angle of all kinds of micelles was smaller than that measured for pure water (Table 2). A hydrophilic and neutral surface has been shown to minimize protein corona formation and extend circulation time following intravenous administration.^45^ The highest contact angle was determined for micelles functionalized with BIO, and the lowest contact angle was observed for FA10, FA25, and FABIO50. Thus, both factors, the type of ligand and its density, influence the surface of micelles determined by zeta potential and contact angle.

The functionality of drug delivery systems is also defined by drug loading and release properties. All kinds of micelles presented a high ability of Dtx incorporation (Table 3). The micelles with higher ligand content characterized increased EE and LC of Dtx, and the highest Dtx loading capacity showed BIO50, FABIO50, and FA50. The release process was similar for all kinds of micelles and provided Dtx elution for 168 h (Figure 2).

The cytocompatibility of all kinds of micelles was confirmed in a study conducted according to the ISO 10993-5 and ISO 10993-12 standards. The micelles did not affect the fibroblasts’ viability at the whole studied concentration range, as shown in Figure 4. The next step was evaluation of the internalization of the selected NPs (without ligand, FA10, BIO10, and FABIO10) with encapsulated FITC by cells with high expression of receptors for FA and BIO (HeLa and 4T1) and cells FAR- and SMVT-negative (WI-38 and B16-F10).^35^ The study revealed that the micelles are preferentially internalized by 4T1 and HeLa cells. Significantly lower uptake was observed in B16-F10 and WI-38 (Figure 3). The study showed also that single-targeted micelles were internalized with higher efficacy compared to dual-targeted micelles (FABIO10).

Analysis of cytotoxicity of the NPs was conducted for native Dtx and all kinds of single- and dual-targeted micelles to discover the effect of the type of ligand, number of ligands, and their density on the cytotoxic effect of the delivered drug. For this purpose, 4 types of cancer cells were selected for the study, 2 with high expression of FAR and SMVT (HeLa and 4T1) and, for comparison, 2 types of cells which present high expression of SMVT and very low expression of FAR (human breast cancer cells, MCF-7 and SK-BR-3). In fact, differences in the cytotoxic effect were observed (Figures 5 and 6). In the case of SK-BR-3 and MCF-7 cell lines, a significantly stronger cytotoxic effect of Dtx delivered in micelles with a higher density of FA and FABIO was observed. In the case of micelles decorated with BIO, the highest potential was exhibited by micelles with an average amount of ligand (BIO25). A significantly different pattern of action of micelles decorated with FA and/or BIO was observed in cells with higher expression of FAR (Figure 6). In the case of HeLa and 4T1 cells, a significantly stronger effect of Dtx delivered in FA-targeted micelles was observed compared to the BIO- and FABIO-targeted micelles. However, the effect of ligand density was varied in HeLa and 4T1 cells. In HeLa cells, a significantly stronger effect of Dtx delivered in micelles with the lowest density of ligand was observed. Interestingly, dual-targeted micelles showed less significant effect compared to single-targeted NPs, especially those decorated with FA. This may be caused by the steric hindrance and difficulties in the binding of ligands to specific receptors.^49^ For more detailed analysis, two options were compared: delivery of Dtx in dual-targeted micelles, where both ligands exist on the same NP (FABIO10), or delivery in a mixture of single-targeted micelles (FA10 + BIO10). The viability of HeLa and 4T1 cells in the presence of a mixture of single-targeted micelles (FA10 + BIO10) was significantly decreased compared to the FABIO10 and other kinds of targeted micelles. This outcome suggests also that the delivery of cytostatic drugs in a mixture of various NPs differing in type of ligand may be very advantageous, and this solution may have the potential to fulfill the challenges related to tumor heterogeneity. Recently, it has been reported that dual-targeted polymersomes, in which two kinds of ligand are present on the same polymersome, were more efficient against OVCAR-3 cells than the mixture of single-targeted polymersomes.^42^ Our results showed the opposite effect, which indicates that the dual-targeting issue is very complex, affected by several factors such as the type of NPs, their morphology, the density of ligands, and the model of cells used in the study.^50,51^

In the case of 4T1 cells, there was no significant effect of ligand density (Figure 6), although a slightly stronger effect was observed for Dtx encapsulated in FA10 and a mixture of FA10 + BIO10 micelles. This may be caused by high resistance of 4T1 cells to Dtx.^47^ In fact, in accordance with the published data, significantly higher doses of Dtx were used for 4T1 cells.^52,53^ Additional analysis was conducted with the use of cytometry technique to determine the difference in cytotoxic effect between Dtx delivered in FABIO10 or FA10 + BIO10 micelles. It was shown that Dtx in both studied experimental formulations (FABIO10 and FA10 + BIO10) exerts high cytotoxic activity and induces morphological changes in 4T1 cells (Figure 7). Moreover, it was found that 1.28 μM of Dtx encapsulated in FABIO10 and a mixture of FA10 + BIO10 micelles affects cell cycle distribution and induces i/apoptosis (shown as an increase in the number of depolarized cells, DNA fragmentation induction, and activation of Annexin V), as well as ii/redox imbalance in the studies of breast cancer cells (Figure 8).

In vivo studies indicated the specificity of micelle binding to tumors with overexpression of FA and BIO receptors (murine breast cancer-4T1) (Figure 9A). Interestingly, no increase in the efficiency of dual-targeted micelle binding was observed, and even their binding efficiency was at a similar level as that of micelles without ligands. In the case of tumors with low expression of FA and BIO receptors (murine melanoma—B16-F10), no increased binding of micelles to tumors was observed (Figure 9B).

In the last stage of the research, therapeutic experiments were conducted by using the tested micelles with Dtx. The experiments were conducted on two murine models differing in the expression of the tested receptors: breast cancer with high expression and melanoma with weak expression. The obtained data indicate the specificity of single-targeted (FA10) and mixtures of FA10 + BIO10 micelles binding to tumors with overexpression of FA and BIO receptors in the case of breast cancer (Figure 10 A). However, no such relationship was observed for melanoma tumors. Similar to experiments examining micelle internalization into tumors, we observed a reduced therapeutic efficacy of dual-targeted micelles in murine breast cancer (Figure 10B).

The conducted research enabled a better understanding of the behavior of micelles single- and dual-targeted with FA and/or BIO. Although the dual-targeted micelles in which two ligands are present on the same NP (FABIO) appeared less effective against cancer cells compared to single-targeted micelles, the highest therapeutic potential was demonstrated for a mixture of two kinds of single-targeted micelles (FA10 + BIO10). This solution seems reasonable due to avoiding steric hindrance but enabling targeting of two different receptors. The results of the study may help to improve the specificity of micellar formulation with docetaxel in cancer therapy.

## 5 Conclusion

There is a growing interest in using the combination of FA and biotin (BIO) as targeting ligands because dual-targeted NPs are expected to overcome the problem associated with the toxicity of conventional chemotherapy and with tumor heterogeneity. The presented study was focused on a comprehensive analysis of the effect of the type and amount of targeting ligand (FA and/or BIO) on the morphology of micelles, their physicochemical properties, drug loading and release properties, cell internalization, in vitro and in vivo cytotoxicity against several kinds of cancer cells.

It has been found that the morphology of micelles was mainly controlled by the PLA–PEG polymer because it did not differ between all kinds of single- and dual-targeted micelles. All kinds of micelles were characterized negative zeta potential, which is advantageous for the stability of NPs after intravenous injection. The micelles with a higher ligand content were characterized by increased Dtx loading properties. The release process was similar for all kinds of micelles and provided drug delivery for 168 h. The cytocompatibility of all kinds of drug-free single- and dual-targeted micelles was confirmed against human fibroblasts. The in vitro cytotoxicity study of single- and dual-targeted Dtx-loaded micelles demonstrated that their effect strongly depends on the type of cancer cells and their surface characteristics. The micelles with a higher density of ligands may have higher efficiency against cells with lower expression of cell surface receptors (SK-BR-3 and MCF-7). The highest cytotoxic effect against SK-BR-3 and MCF-7 cells was exhibited by Dtx delivered in FA50 micelles, slightly lower in BIO25 and FABIO50. In the case of cancer cells with overexpression of receptors for FA and BIO (HeLa and 4T1), micelles with a lower density of ligands showed better efficiency, especially FA10. However, in all cases, single-targeted NPs appeared to be more effective compared to the dual-targeted micelles. Also, the confocal microscopy analysis enabled us to observe preferential internalization of single-targeted micelles by cells that characterize high expression of FAR and SMVT (4T1 and HeLa) compared to the FAR- and SMVT-negative cells (B16-F10 and WI-38). Therefore, an additional study was conducted to compare the mixture of single-loaded micelles (FA10 + BIO10) with that of dual-targeted micelles (FABIO10). In vitro analysis showed a higher cytotoxic effect of Dtx delivered in a mixture of FA10 + BIO10 micelles against HeLa and 4T1 cells. Finally, the therapeutic efficacy of the constructed Dtx-loaded micelles was verified in the mouse model of 4T1 breast cancer and B16 – F10 melanoma. The most significant tumor growth inhibition in the 4T1 breast cancer model was observed for Dtx delivered in FA10 + BIO10 and slightly lower for Dtx in FA10 micelles. In B16-F10 melanoma tumors, which were characterized by low expression of both receptors, no differences in the tumor growth inhibition were observed.

The conducted research enabled a better understanding of the influence of the type, number, and density of ligands on various cells differing in the receptors’ pattern. The results may also help to improve the specificity of micellar formulation with docetaxel in cancer therapy.

## Supplementary Material



## Data Availability

The data that support the findings of this study are available from the corresponding author upon reasonable request.

## Notes

Cryo-TEM images of drug-free PLLA–PEG micelles and PLLA–PEG micelles loaded with docetaxel; comparison of ^1^H NMR spectra of FA, PLLA–PEG micelles, and PLLA–PEG micelles functionalized with folic acid (FA10, FA25, and FA50); comparison of ^1^H NMR spectra of biotin, PLLA–PEG micelles, and PLLA–PEG micelles functionalized with biotin (BIO10, BIO25, and BIO50); comparison of ^1^H NMR spectra of FA, biotin, PLLA–PEG micelles, and PLLA–PEG micelles functionalized with folic acid and biotin (FABIO10, FABIO25, and FABIO50); comparison of IC_50_ (nM) values depending on the formulation of docetaxel and cell line; and comparison of fluorescence emission value for various kinds of FITC-loaded micelles (PDF)
