# Supplementary material for: Comparison of Micelles Single- and Dual-Targeted with Folic Acid and Biotin as the Delivery System of Docetaxel—The Influence of the Type and Amount of the Ligand on Morphology, Physicochemical Properties, and Cytotoxicity
Source: Mol Pharm. 2025 Jun 26;22(8):4662–78. doi: 10.1021/acs.molpharmaceut.5c00215 (PMC12326352; doi:10.1021/acs.molpharmaceut.5c00215)
Supplement: Supplementary file 1 [file mp5c00215_si_001.pdf]

Supplemetary materials

Comparison of micelles single- and dual-targeted with folic acid and biotin as delivery system of docetaxel - the influence of type and amount of ligand on morphology, physicochemical properties and cytotoxicity

Magdalena Jurczyk[1,2], Ryszard Smolarczyk[3], Monika Musiał-Kulik[2], Joanna Ciepła[3], Sybilla Matuszczak[3], Tomasz Cichoń[3], Justyna Czapla[3], Marcelina Bochenek[2], Aleksander Foryś[2], Dorota Wrześniok[1], Artur Beberok[1], Katarzyna Jelonek[2*]

[1] Department of Pharmaceutical Chemistry, Faculty of Pharmaceutical Sciences in Sosnowiec, Medical University of Silesia, Jagiellońska 4, 41-200 Sosnowiec, Poland

[2] Centre of Polymer and Carbon Materials, Polish Academy of Sciences, Curie-Skłodowska 34 St., 41-819 Zabrze, Poland

[3] Center for Translational Research and Molecular Biology of Cancer, Maria Skłodowska-Curie National Research Institute of Oncology, Gliwice Branch, Wybrzeże Armii Krajowej Street 15, 44-102 Gliwice, Poland

* Corresponding author: kjelonek@cmpw-pan.pl (Katarzyna Jelonek)

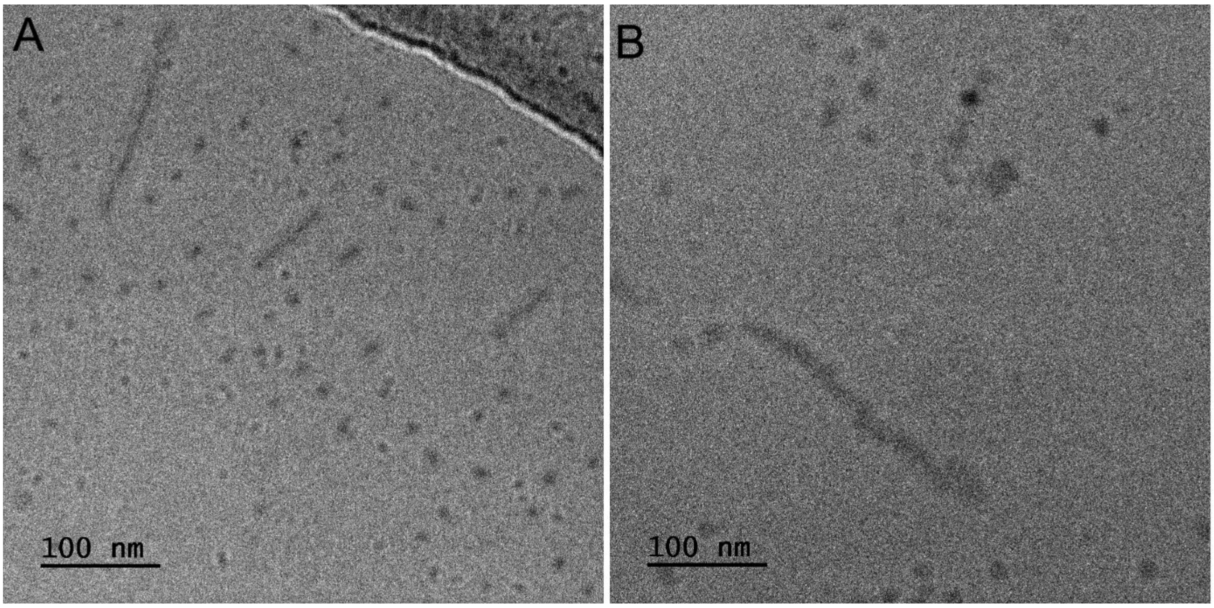

Figure 1S. Cryo-TEM images of drug-free PLLA-PEG micelles (A) and PLLA-PEG micelles loaded with docetaxel (B).

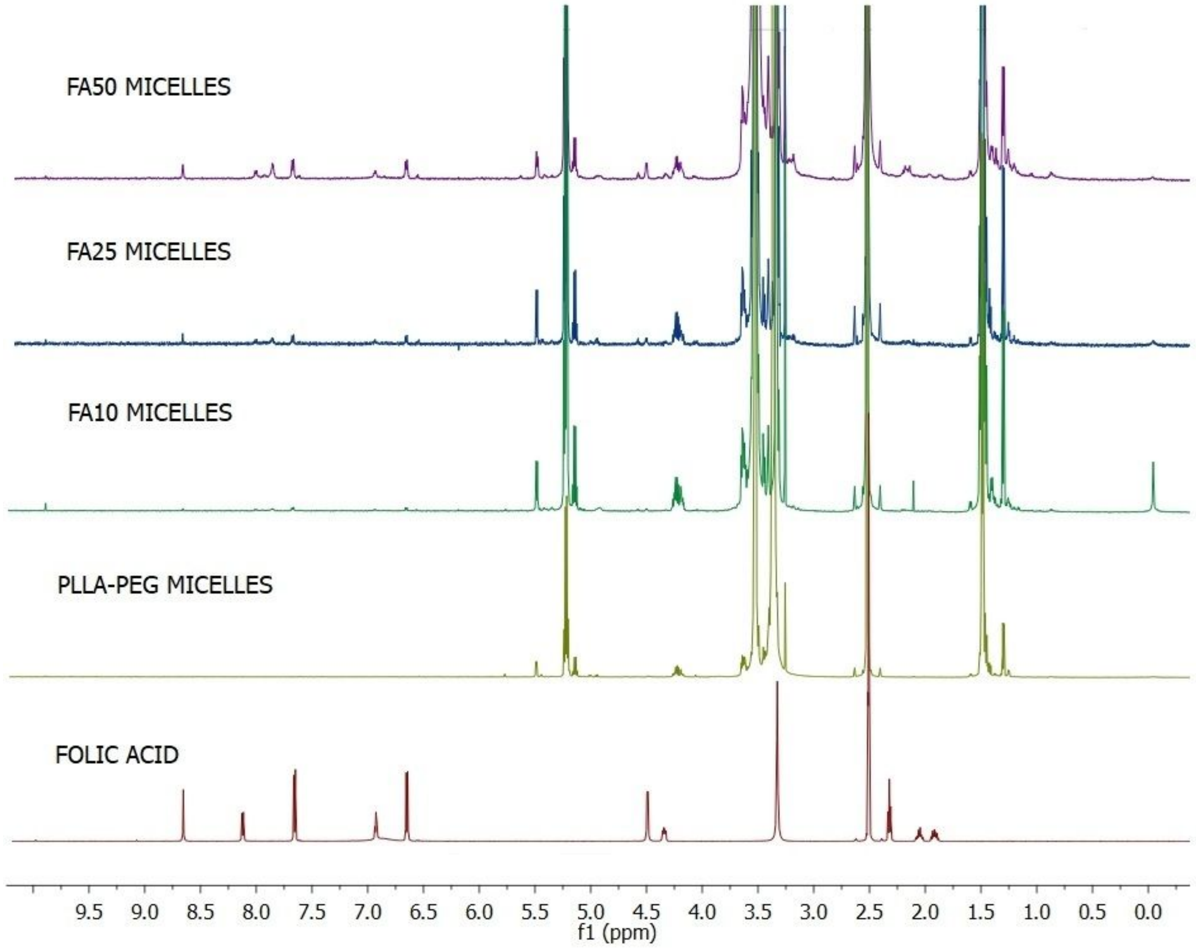

Figure 2S. Comparison of ^{1}H-NMR spectra of folic acid, PLLA–PEG micelles and PLLA-PEG micelles functionalized with folic acid (FA10, FA25 and FA50).

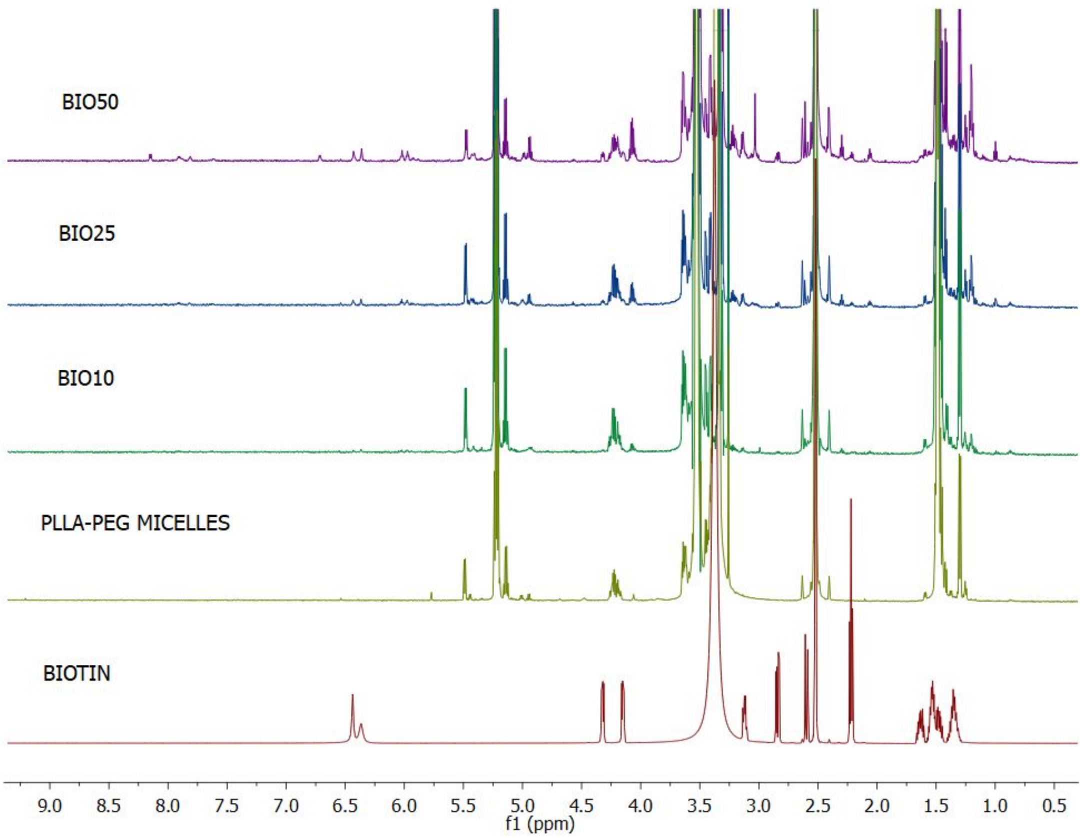

Figure 3S. Comparison of ^{1}H-NMR spectra of biotin, PLLA–PEG micelles and PLLA-PEG micelles functionalized with biotin (BIO10, BIO25 and BIO50).

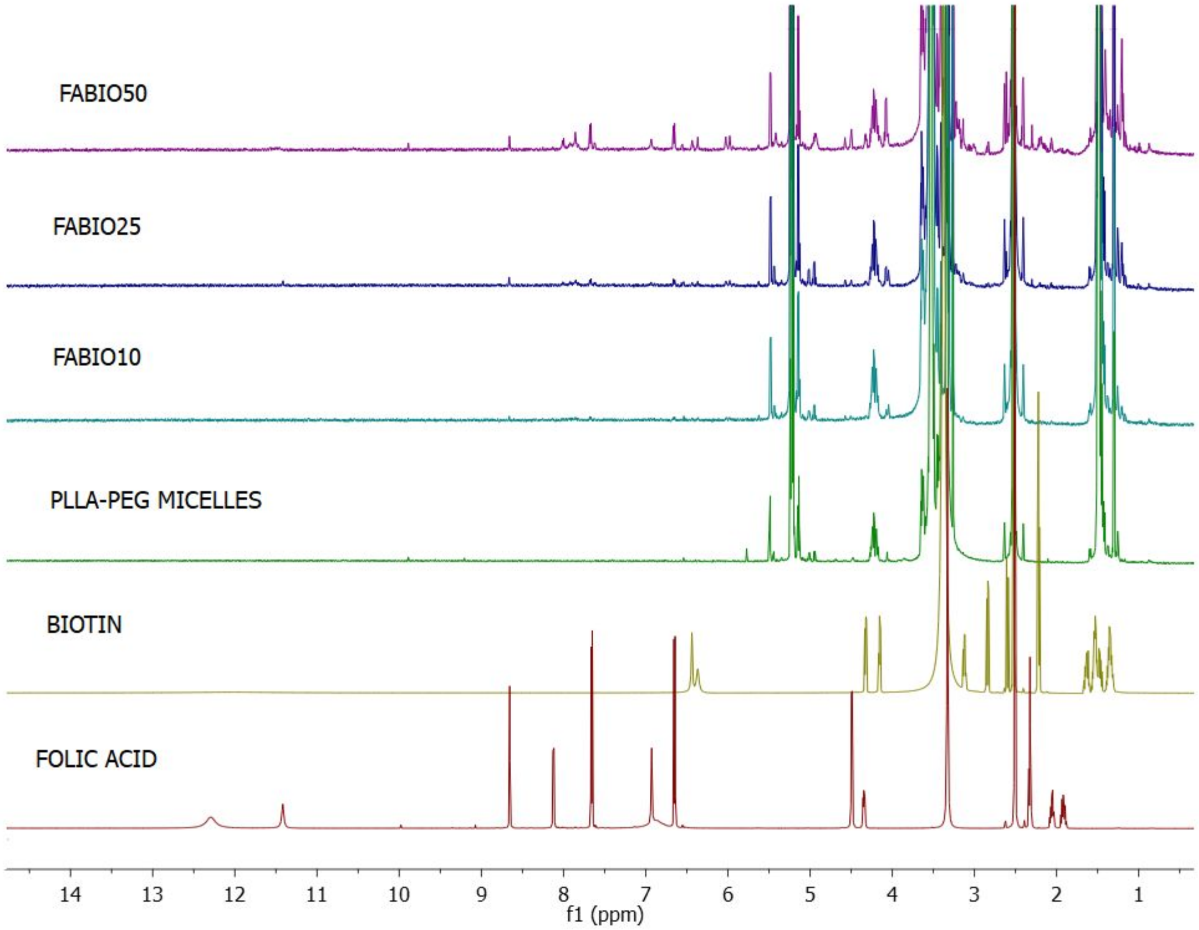

Figure 4S. Comparison of ^{1}H-NMR spectra of folic acid, biotin, PLLA–PEG micelles and PLLA-PEG micelles functionalized with folic acid and biotin (FABIO10, FABIO25 and FABIO50).

Table 1. Comparison of IC_{50} (nM) values depending on formulation of docetaxel and cell line.

	**SK-BR-3**	**MCF-7**	**HeLa**	**4T1**
**Dtx**	4.6	2.1	1.0	101.1
**FA10**	343.6	> 1280	33.9	197.7
**FA25**	462.4	309.3	83.9	532.2
**FA50**	43.9	163.2	64.9	556.2
**BIO10**	159.8	369.7	141.8	850.9
**BIO25**	102.8	286.7	193.7	877.8
**BIO50**	273.7	> 1280	186.5	1048.5
**FABIO10**	660.3	> 1280	81.5	3368.2
**FA10+BIO10**	-	-	10.0	567.9
**FABIO25**	691.3	312.4	144.1	1003.2
**FABIO50**	431.2	165.5	681.7	1034.2

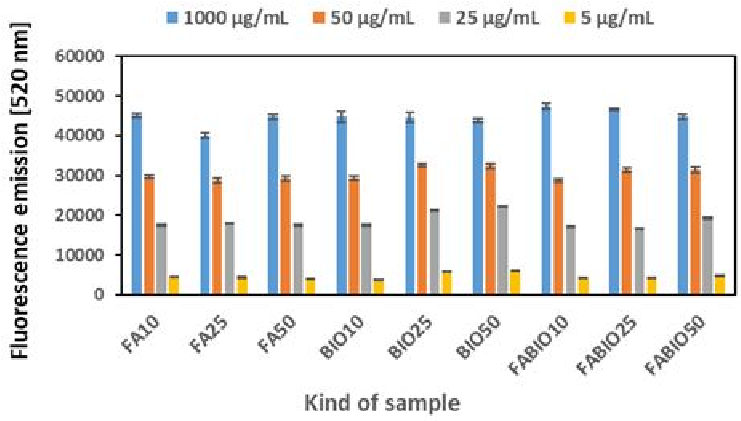

Figure 5S. Comparison of fluorescence emission value for various kinds of FITC-loaded micelles.
